# Acceptability of home-based HPV self-sampling for cervical cancer screening among users and providers in the West region of Cameroon: a cross-sectional study

**DOI:** 10.1186/s12913-025-13467-1

**Published:** 2025-10-03

**Authors:** Alida Manoëla Datchoua Moukam, Nasteha Salah, Gilles W. Tankeu Happi, Loïc D. Djommo Metchehe, Sophie Lemoupa Makajio, Ania Wisniak, Jessica Sormani, Bruno Kenfack, Pierre Vassilakos, Antoine Socpa, Patrick Petignat, Nicole C. Schmidt

**Affiliations:** 1 Institute of Global Health, Faculty of Medicine, University of Geneva, 9 Chemin des Mines, Geneva, 1202 Switzerland; 2 Gynaecology Unit, Department of Women, Children and Adolescent, University Hospitals of Geneva, Geneva, Switzerland; 3 RSD Institute, Yaounde, Cameroon; 4 Department of Gynaecology and Obstetrics, Regional Hospital of Bafoussam, Bafoussam, Cameroun; 5 Global Research Agency, Dschang, Cameroon; 6 Geneva School of Health Sciences, HES-SO University of Applied Sciences and Arts Western Switzerland, Geneva, Switzerland; 7 Department of Gynaecology and Obstetrics, Dschang Regional Annex Hospital, Dschang, Cameroon; 8 Department of Obstetrics and Gynaecology, Faculty of Medicine and Pharmaceutical Sciences, University of Dschang, Dschang, Cameroon; 9 Geneva Foundation for Medical Education and Research, Geneva, Switzerland; 10 Faculty of Arts, Letters and Social Sciences, Department of Anthropology, University of Yaounde I – Lab. CASS-RT, Yaounde, Cameroon; 11 Department of Paediatrics, Gynaecology and Obstetrics, Faculty of Medicine, University of Geneva, Geneva, Switzerland; 12 Faculty of Social Science, Catholic University of Applied Science, Munich, Germany

**Keywords:** Acceptability, Cervical cancer screening, Home-based HPV self-sampling, Quantitative study, sub-Saharan Africa

## Abstract

**Background:**

The World Health Organization’s 90-70-90 goal aims to eliminate cervical cancer (CC) as a public health issue, with a target of up to 70% of women screened by 2030. However, many countries, including Cameroon, are far from achieving this goal. Home-based human papillomavirus (HPV) self-sampling is a promising approach to improve participation rates. The main objective of this study was to explore the acceptability and feasibility of home-based HPV self-sampling in the West region of Cameroon.

**Methods:**

A quantitative, descriptive, cross-sectional study conducted between January 11 and February 05, 2024, was embedded in a cluster-randomised controlled trial comparing home- vs. hospital-based CC screening. Women eligible for CC screening, male partners or close relatives, community leaders, and healthcare professionals (HCPs) living in Dschang health district responded to a structured questionnaire. Simple and multivariate analyses were performed to assess the association between acceptability of home-based HPV self-sampling, CC screening practices, and sociodemographic factors such as education, professional status, residence, and income. Preferences for implementation (e.g., seasonal timing) were also explored.

**Results:**

A total of 556 participants (300 women, 70 male partners, 153 HCPs, and 33 community leaders) were recruited. Overall, 77.5% of participants expressed favourable attitudes toward home-based HPV self-sampling, with acceptability rates of 73.7% for women, 65.7% for men, 90% for community leaders, and 87.6% for HCPs. Previous CC screening was reported by 33% of women, and was significantly associated with being over 40 years old (aOR = 2.1, *p* = 0.007), with a monthly income of > 50,000 XAF (aOR = 2.2, *p* = 0.049), and having good knowledge of CC (aOR = 2.6, *p* = 0.001). Morning screening implementation was preferred by most participants, with 60% favouring year-round screening. More than 70% preferred face-to-face communication of HPV test results from SMS or phone calls, at hospitals for women (63%), men (50%), and HCPs (65.4%); while community leaders preferred home disclosure.

**Conclusion:**

Home-based HPV self-sampling was highly accepted across all study groups and was unaffected by sociodemographic factors. Previous screening practices were associated with age, income, and knowledge. Home-based HPV self-sampling screening can address access disparities to CC screening. Community involvement in planning and implementing these programs is essential to ensure their success.

**Trial registration:**

Ethical Cantonal Board of Geneva, Switzerland (CCER, N°202100085), (ClinicalTrials.govID NCT06166420 / 20231204) and the National Ethics Committee for Human Health Research in Cameroon (N°2023/09/1579/CE/CNERSH/SP).

**Supplementary Information:**

The online version contains supplementary material available at 10.1186/s12913-025-13467-1.

## Background

Cervical cancer (CC) ranks fourth among all cancers in incidence and mortality worldwide. It is currently the leading cause of cancer deaths for women in 38 countries [1] with approximately 660 000 new cases and 350 000 deaths in 2022 [2]. This figure is likely to evolve, particularly in vulnerable communities [1]. CC is highly preventable and can be easily treated if detected at early stages [3].

The World Health Organisation’s global strategy defines elimination as reducing the number of annual new cases to 4 per 100 000 women or less; and sets three goals to be achieved by 2030 in order to take all countries on the pathway to elimination in the coming decades: 90% of girls vaccinated against human papillomavirus (HPV) by age 15, 70% of women by age 30–49, screened with a high-quality and high-performance test; and 90% of women with cervical disease receiving treatment [1–4].

In Cameroon, a lower-middle-income country, CC is the second leading cause of cancer-related deaths among women after breast cancer [5]. Only 3–5% of eligible women are screened, and there is no effective national cervical cancer prevention program [4, 6]. Cameroon has a population of 8.02 million women aged 15 or more who are at risk of developing CC. Current estimates indicate that every year, 2770 women are diagnosed with CC and 1787 die from the disease [7, 8]. Thus, CC is ranked 2nd most frequent cancer among women in Cameroon [7, 8].

To reduce the burden of this disease, a 5-year CC screening program was introduced in 2018 at Dschang District Hospital in Cameroon [9]. This program aimed to prevent CC among women aged between 30 and 49 years. It provided free CC screening and treatment of precancerous lesions in a single visit (“screen and treat” approach). More than 6,500 women were enrolled in the said program. However, since recruitment was initially slower than expected, the reasons for the delay were explored. Previous studies identified barriers such as long distances from the few existing health facilities, prohibitive transportation costs, and lack of time to report for screening [10]. Other obstacles to CC screening highlighted by the international literature include self-assertion of being healthy or fear of screening, perceived negative attitudes towards patients, long waiting times, and lack of male support [11–14]. Furthermore, scepticism due to previous sad experience at hospitals [10] and limited physical access to screening centres, due to impracticable roads, were mentioned [15, 16]. To overcome barriers such as distance, cost, and lack of time, Arrosi et al. showed in an Argentinian study that self-collection of samples for HPV testing by community health workers during home visits resulted in a four-fold increase in screening uptake, supporting the idea that home-based HPV self-sampling may contribute to a significant increase in coverage [17]. In Ethiopia, Brandt et al. argued that home-based self-sampling for CC screening is a socially acceptable and feasible “task-shifting” method that may increase CC screening access and coverage [18].

Therefore, we hypothesized that home-based HPV self-sampling may improve the acceptance of CC screening in Cameroon, where distance, cost, and lack of time are important barriers to CC screening [10, 12]. Thus, our aim was to quantitatively assess the acceptability of home-based HPV self-sampling among women, male partners, community leaders, and HCPs in settings where this approach has not yet been implemented. Furthermore, the study sought to identify factors influencing women’s decision to participate in CC screening, and to explore the key elements valued by different stakeholders to support the effective implementation of home-based HPV self-sampling.

## Methods

### Study design

This study (referred to as study B; Fig. 2) is part of a 2-arm randomized-controlled study (study A) registered on (ClinicalTrials.govID NCT06166420 / 2023-12-04). We aimed at exploring whether HPV self-sampling is as feasible and effective in a home/community-based setting as in a standard hospital setting. Study A compared HPV self-sampling performed at hospital to home-based setting. Study B, which was rolled out prior to study A in areas where home-based HPV self-sampling was not provided, aimed at assessing the acceptability and preferences for the implementation of HPV home-based self-sampling, within different population groups, using a quantitative approach. The study used a descriptive cross-sectional design with an analytical component. The descriptive aspect focused on the acceptability and preferences for the implementation of HPV self-sampling at home in the four study groups. The analytical component investigated potential parameters associated with screening. Predictors included sociodemographic characteristics (age, income, and marital status), perceptions of screening (knowledge and attitudes), and contextual barriers (distance and accessibility). Multivariate models were applied to assess associations between these variables and screening uptake. Results were used to identify key determinants and providing a foundation for tailoring intervention strategies. We used the STROBE reporting guidelines.

### Setting

**Fig. 1:**
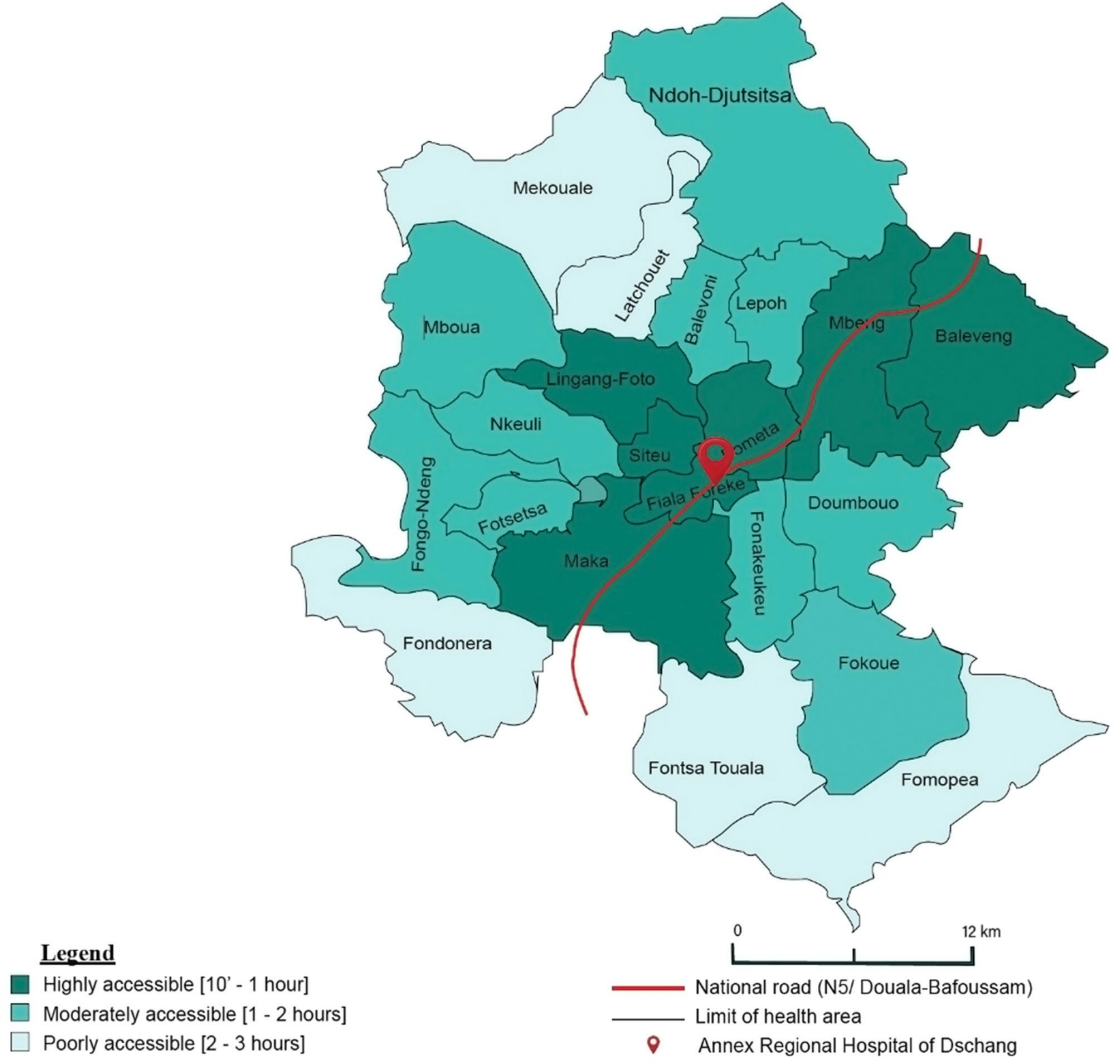
Accessibility to the health areas of Dschang Health District, in terms of travel time from Fiala Foreke, considered as “central health area” *(map adapted from the Ministry of Public Health – Cameroon)*

**Fig. 2:**
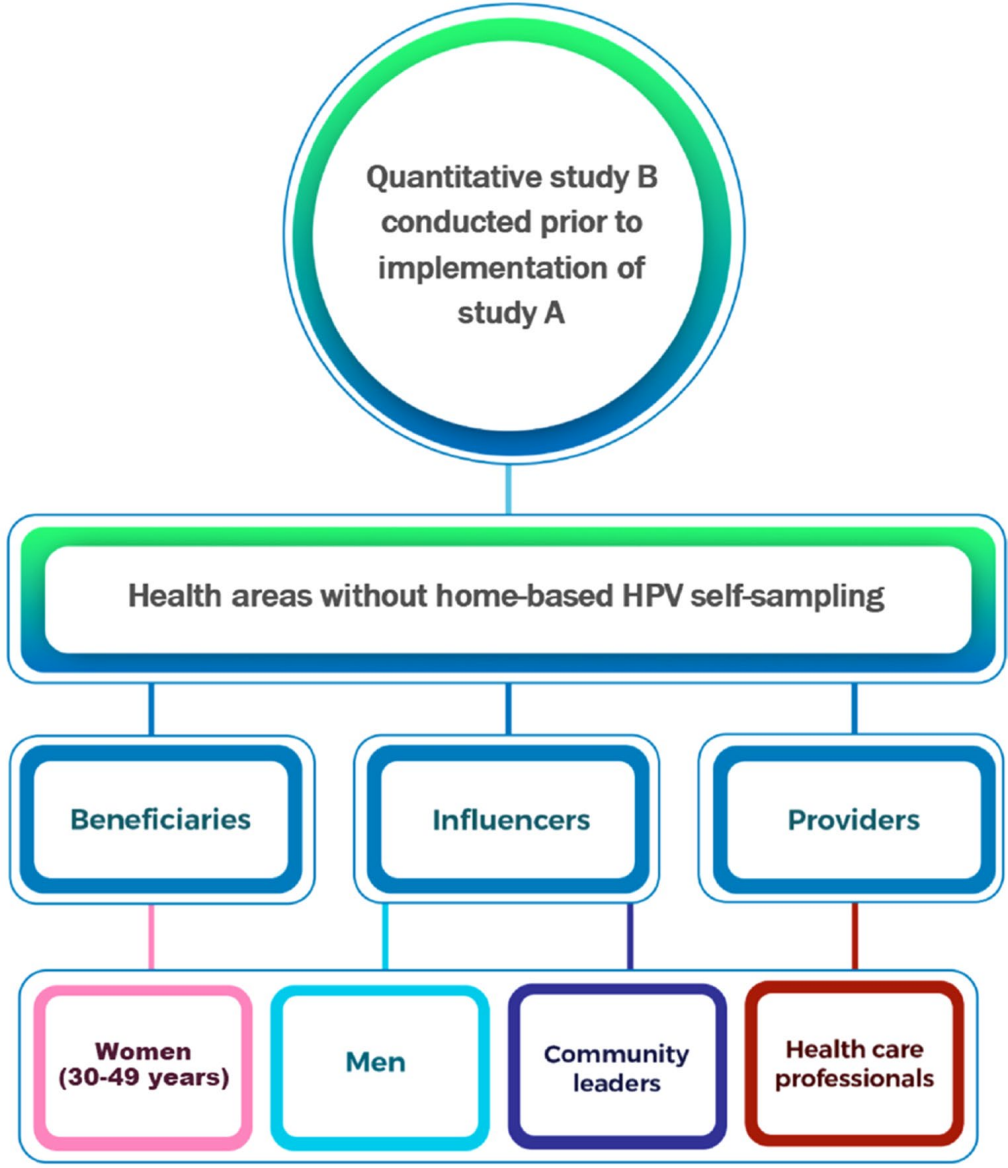
Flow chart of study B (Acceptability study)

This study was conducted in Dschang Health District (DHD) (Fig. 2), which is divided into 22 health areas. DHD covers an area of approximately 1,060 km² and has approximately 236 500 inhabitants. Women at childbearing age represent nearly one quarter of the population. The district has a public university, private university, 66 secondary schools, and 282 primary schools. Most of which are located in urban areas. In terms of healthcare, there are 72 health facilities, comprising 44 public and 28 private. These range from the Cameroonian health system category 3 (Dschang Regional Annex Hospital) to category 6 (integrated health facilities). Accessibility varies from one health area to the other, and is classified according to travel time (by motorcycle) from the health district office in Fiala-Foreke to each area’s main health facility; considering road conditions during the rainy season [19]. Areas are categorized as poorly accessible (2 to 3 h travel time) or moderately to highly accessible (see Fig. 1). CC screening is currently offered only in hospitals throughout the West Region of Cameroon. Home-based HPV self-sampling is still under evaluation and not yet part of routine care.

The figure illustrates the geographical layout of DHD and its health areas. Accessibility is expressed in terms of time. The most accessible health area (Siteu, Fometa) is 10 min from the district office, while the least accessible areas, including Fondonera, Mekouale, and Fomopea, are approximately 3 h away.

### Participants

Data were collected between January and February 2024, from nine health areas within the Dschang Health District, including one urban, two semi-urban, and six rural areas. The study included four groups of stakeholders: women eligible for CC screening aged 30–49 years who were potential beneficiaries; and stakeholders who might influence women’s decision processes, such as husbands or close relatives and community leaders (see Fig. 2); and HCPs, who serve as potential providers of CC screening. Stakeholders were defined as an ‘individual or a group responsible for, or affected by health-and healthcare‐related decisions that can be affected by research evidence’ [20]. Further details are provided in Table 1.

**Table 1:** Participants’ characteristics

Criteria	Women	Men / Relatives	Community leaders	Health care professionals
Inclusion criteria	Having lived in Dschang Health District (DHD) for ≥ 12 months			Working in a health facility in DHD as an accredited HCP
	Aged 30–49 years	Partner or close relative living in the same house		
Exclusion criteria	Previous HPV-home self-sampling test			Works in a “red zone” (insecure border area with the South-western region of Cameroon)
				Staff of the 3T research program
	Residing in a health area in study A			
	Existing mental/cognitive limitation (either medically diagnosed or witnessed by family/friends)			
	Unavailability after three appointments			

### Variables

In accordance with the study objectives, the main outcomes were the acceptability of home-based HPV self-sampling, practice of CC screening, and preferences for the implementation of home-based HPV self-sampling. The predictors were sociodemographic parameters (such as age, instruction level, place of residence, monthly income, and matrimonial status), knowledge of CC, and previous CC screening.

The data comprise both quantitative and qualitative variables. Age, originally quantitative, was later categorized as 30–40 years and 40–49 years for the women; <30 years, [30–40] years and ≥ 40 years for health professionals. Monthly income for women and households was recoded for predictive analysis into < 50,000 Central African XAF franc (FCFA)/≥50,000 XAF and ≤ 100,000 XAF/>100,000 XAF respectively, for the purpose of predictive analysis. The minimum wage in Cameroon is 41,875 XAF (approximately 65 euros) [21]. Health areas were recorded according to the distance from the main office of the DHD in zones 1”, 2, and 3”. Duration of residence in the health area was classified into < 3 years, [3–10] years and > 10 years. Instruction levels were categorized as “has never been to school”, “primary” (having completed any class at primary level), “secondary” (having completed any class at secondary level), and “higher” (having completed any class at higher level). Employment status was grouped into “formal sector”, “informal sector”- referring to a wide range of activities and businesses outside formal economy, operating independently and often characterized by low wages, job insecurity, and absence of legal protection [22] - and “no income/joblessness”. Knowledge of CC was assessed by 8 questions scored 1 for correct and 0 for incorrect answer. Scores ranged from 0 to 8 and were categorized as “good” (≥ 4/8) or “insufficient” (< 4/8) based on a 50% cut-off. Acceptability of home-based HPV self-sampling was measured through 9 to 10 questions. They were tailored for the four groups, using a 5-item Likert scale: “totally disagree”, “disagree”, “neutral”, “agree” and “totally agree”. “Agree” and “totally agree” (for positive formulated questions), or “not agree” and “totally disagree” (for negative formulated questions) rated “1”for each answer. Scores were summed and dichotomized as “favourable” or “unfavourable”, using a 50% threshold. Preferences regarding feasibility and implementation of home-based HPV self-sampling were analysed descriptively and expressed in terms of responses proportions.

### Data collection

Data were collected by a team of trained data collectors through a pretested questionnaire (additional file 1) for each target group. The questionnaire comprised four sections: sociodemographic information, knowledge about CC, acceptability of home-based HPV self-sampling, and feasibility of its implementation. Data were digitized using a mobile data collection tool (KoboToolbox). And constantly monitored and supervised by the data manager. Participants did not receive any incentives for their involvement in the study.

### Bias

Before conducting the study, potential sources of bias were carefully addressed. A pretested questionnaire was used. Selection bias was minimized by recruiting participants exclusively from areas not involved in the randomized controlled trial (Study A). Health areas with diverse accessibility levels were included. To reduce information bias, data collectors underwent a three-day training session to ensure that the questions were phrased neutrally, so as to minimize unintentional influence and missing data. Data collectors, were provided with a “Data Collector’s Guide”, specifically created for the study. They were closely supervised by a data manager.

### Study size

Sample sizes for women and HCPs were calculated using the OpenEpi platform, based on various parameters. These include population size, assumed frequency of acceptance of home-based HPV self-sampling, a 95% confidence interval, and a 5% margin of error. This resulted in an estimated sample size of 287 women and 141 HCPs. Assuming a 1:1 ratio between women and their partners or close relatives (i.e., one partner per woman surveyed). The estimated sample size for partners/close relatives was set at 287. Due to the absence of preliminary data on community leaders (traditional, social, and religious), we relied on contextual knowledge and applied a quota sampling approach for this group. Assuming that, three community leaders (traditional, social, and religious^1^) per health area, a total sample of 27 of them were to be selected from the nine health areas.

### Statistical methods

Qualitative variables were summarized as absolute counts and percentages. Quantitative variables were presented as means and standard deviations. Simple and multivariate logistic regression analyses were performed to assess the association between outcomes and exposure. Multivariate models included variables with a p-value < 15% from univariate analysis, as suggested by a simulation by Bursac et *al.* [23]. Data management and preliminary analyses were performed using MS Excel 2016. We analysed the data using both descriptive and analytical approaches, using IBM SPSS 25. The threshold for statistical significance was set at 5%.

## Results

### Sociodemographic characteristics of participants

A total of 556 participants (300 women, 70 male partners, 153 HCPs, and 33 community leaders) were recruited from the nine health areas (Table 2). Thus, the target sample size was attained in three groups out of four. However, we could only recruit one quarter of women’s partners or close relatives. The main reason being the absence of partners for professional or family related occupations. Moroever, 15% of women live without partners.

The mean age (± standard deviation) was 37.9 (± 5.9) years for women, 51.8 (± 14.3) years for men, 33.6 (± 8.7) years for HCPs, and 54.1 (± 14.9) years for community leaders. Participants had been living in their respective communities for less than 3 years (24.5%) and more than 10 years (33.1%). Women, their partners/close relatives, and HCPs mostly lived in Fiala Foreke urban health area, while community leaders were mostly found in rural areas.

The majority of participants (≥ 61% among all groups) were in a relationship. Furthermore, monotheism was the main practiced religion by women (80.4%), men (84.1%), HCPs (91.5%). Whereas community leaders identified themselves mostly as animists (36.4%) or monotheists (36.4%).

Secondary education was the predominant level of instruction for women and men. While, higher education (defined as having started at least one school term of tertiary education or pursuing professional training after secondary education) was more common among community leaders (42%) and HCPs (70%).

Regarding employment, women predominantly worked in the informal sector (73.0%), while men were mainly employed in the formal sector (78.3%).

Monthly income levels were significantly associated with formal or informal sector of activity (*p* < 0,001). Women (84.7%), men (65.7%) reported a monthly income of ≤ 50,000 XAF, and same for HCPs (44.4%).

**Table 2:** Sociodemographic characteristics

Sociodemographic characteristics	Women		Men		Healthcare professionals		Community leaders	
	*n*	(%)	*n*	(%)	*n*	(%)	*n*	(%)
**Place of residence**								
Urban	140	(46.7)	29	(41.4)	92	(60.1)	9	(27.3)
Semi-urban	29	(9.7)	25	(35.7)	21	(13.7)	6	(18.2)
Rural	131	(43.6)	16	(22.9)	40	(26.2)	18	(54.6)
**Age**								
Mean ± SD	37.9 ± 5.9		51.8 ± 14.3		33.6 ± 8.7		54.1 ± 14.9	
**Civil status**								
Single	47	(15.7)	9	(12.8)	60	(39.2)	4	(12.2)
In a relationship	253	(84.3)	61	(87.2)	93	(60.8)	29	(87.9)
**Duration of residence in the health area**								
< 3 years	63	(28.5)	8	(11.4)	58	(37.9)	7	(21.2)
3–10 years	86	(38.9)	10	(14.3)	55	(36.0)	6	(18.2)
> 10 years	72	(32.6)	52	(74.3)	40	(26.1)	20	(60.6)
**Level of instruction**								
Never been to school/primary	94	(31.3)	27	(38.6)	1	(0.7)	8	(24.2)
Secondary	153	(51.0)	34	(48.6)	44	(28.8)	11	(33.4)
Higher	53	(17.7)	9	(12.9)	108	(70.6)	14	(42.4)
**Religion**								
Animism	36	(12.0)	7	(10.1)	09	(5.9)	12	(36.4)
Atheist/others**	23	(7.7)	4	(5.8)	04	(2.6)	1	(3.0)
Monotheism*	241	(80.4)	58	(84.1)	140	(91.5)	12	(36.4)
Missing	-	-	1	-	-	-	8	-
**Monthly income (XAF)**								
≤ 50 000	254	(84.7)	46	(65.7)	68	(44.4)	N/I	
> 50 000	46	(15.3)	24	(34.3)	85	(55.6)		
Not declared	0	(0.0)	4	(5.7)	0	(0.0)		
**Monthly income of the household (XAF)**								
≤ 100 000	177	(59.1)	46	(65.7)	93	(60.8)		N/I
> 100 000	123	(41.0)	15	(21.5)	60	(39.2)		N/I
Not declared	0	(0.0)	9	(12.9)	0	(0.0)		N/I
**Employment**								
Formal sector	68	(22.6)	18	(78.3)		N/I		N/I
Informal sector	219	(73.0)	1	(4.3)		N/I		N/I
No income/joblessness	13	(4.4)	4	(17.3)				
Missing	-	-	47	-				
**Number of children living with the woman**								
≤ 4 children	173	(57.7)		N/I		N/I		N/I
> 4 children	127	(42.3)		N/I		N/I		N/I
**Sex**								
Female		N/I		N/I	108	(70.6)	27	(81.8)
Male		N/I		N/I	45	(29.4)	6	(18.2)
**Level of study of partner**								
Never been to school/primary	59	(23.0)		N/I	6	(5.6)		N/I
Secondary	133	(60.0)		N/I	31	(29.3)		N/I
Higher	64	(25.0)		N/I	69	(65.1)		N/I
Missing	44	-			47	-		
**Occupation of partner**								
Formal sector	86	(33.6)		N/I	70	(66.0)		N/I
Informal sector	157	(61.3)		N/I	27	(25.5)		N/I
No income/joblessness	13	(5.1)		N/I	09	(8.5)		N/I
**Qualification**								
Assistant nurse, Laboratory Technician, Others		N/I		N/I	72	(40.1)		N/I
Nurse		N/I		N/I	57	(37.2)		N/I
Medical doctor		N/I		N/I	11	(7.2)		N/I
Midwife		N/I		N/I	13	(8.5)		N/I
**Years of experience**								
≤ 5 years		N/I		N/I	81	(53.0)		N/I
> 5 years		N/I		N/I	72	(47.1)		N/I
**Community leaders’ role**								
Traditional leader		N/I		N/I		N/I	20	(60.6)
Religious leader		N/I		N/I		N/I	7	(21.2)
Others***		N/I		N/I		N/I	6	(18.2)

N/I: the question was not asked in the target group

XAF: Francs CFA; SD: standard deviation

*all Christians apart from two Muslims among women

**: includes Kamite (01)

***: includes leaders of community associations and traditional healers

### Acceptability of home-based HPV self-sampling

**Fig. 3:**
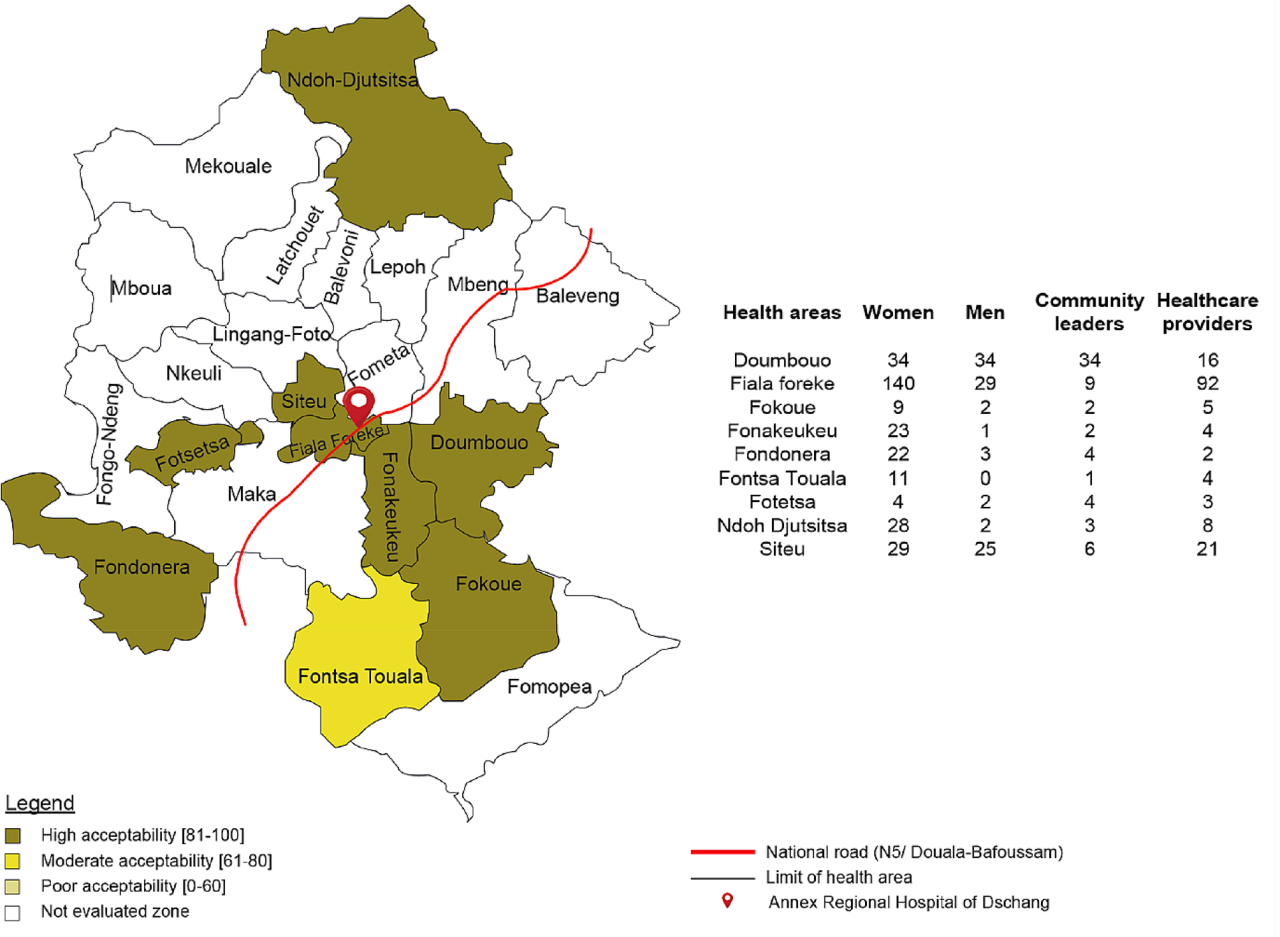
Overall acceptability of home-based HPV self-sampling in Dschang Health District (women, men, community leaders, and healthcare professionals)

The overall acceptability of home-based HPV self-sampling was 77.5% (95% CI: 74.0–81.0%) across all participant groups. Specifically, acceptability rates were 73.7% (95% CI: 68.7–78.6%) among women, 65.7% (95% CI: 54.6–76.8%) among men or close relatives, 87.6% (95% CI: 82.3–92.8%) among HCPs, and 90.9% (95% CI: 81.1–100%) among community leaders. In eight of the nine health areas, acceptability exceeded 81.0%, except for Fontsa Touala, where it was 75% (Fig. 3). More than 80% of women and men reported that the presence of other household members did not hinder the women’s ability to perform HPV self-sampling (86.3% women vs. 88.6% men). However, favourable attitudes were lower among community leaders (57.1%). Confidence in performing HPV self-sampling was expressed by 68.3% of women and 72.9% of men. When asked whether their homes were sufficiently hygienic to conduct the procedure, 56.0% of women, 61.4% of men, and 51.5% of community leaders responded positively. In addition, HCPs demonstrated high confidence in their ability to provide clear explanations about CC and the procedures for home-based HPV self-sampling (90.2%). With nearly all 95.4% reporting they felt adequately trained to counsel women and to help manage HPV-positive results.

### Factors associated with acceptability of home-based HPV self-sampling

None of the sociodemographic factors analyzed significantly influenced home-based HPV self-sampling acceptability (additional file 3). Therefore, additional analyses were conducted to explore the factors influencing whether participants underwent CC screening—either at home or at hospital—referred to here as the “practice of CC screening.”

### Prior practice of CC screening and related knowledge

Among the studied female participants, one woman out of three (*n* = 99) had undergone CC screening at least once. Most of them (76.8%) screened less than 5 years ago (Table 3).

Knowledge of CC was also evaluated. A total of 65% participants had adequate knowledge related to CC. While HCPs showed the highest levels of knowledge (94.1% with adequate knowledge). The proportion of women and male partners or close relatives with adequate knowledge was 62.7% and 64.3%, respectively.

Univariate analysis revealed that women over 40 years (cOR = 1.7 (1.0; 2.8), *p* = 0.035), those with a monthly income above 50,000 XAF (cOR = 2.2 (1.2;4.1), *p* = 0.016), and those with good knowledge of CC (cOR = 2.2 (1.3;3.8), *p* = 0.003) were associated with a higher likelihood of having been screened for CC. Multivariate analysis confirmed these associations. Women aged between 40 and 49 were twice more likely to have undergone CC screening within their lives ([40–50] years: aOR = 2.1 (1.2;3.7), *p* = 0.007) than those aged 30–40. Similarly, participants with good knowledge of CC were significantly more likely to have undergone screening (aOR = 2.6 (1.4;4.7), *p* = 0.001). A monthly income exceeding 50,000 XAF also tended to be associated with higher screening uptake, although this result was borderline significant (aOR = 2.2 (1.0 ;5.0), *p* = 0.049).

**Table 3:** Factors associated with CC screening among women in Dschang health district

		Undergone CC screening				Univariate regression			Multivariate regression		
**Variables**	**N**	Yes		No		cOR	95%CI	p	aOR	95%CI	p
		n	(%)	n	(%)						
**Place of residence**											
Rural	131	38	(29.0)	93	(71.0)	1					
Semi-urban	29	12	(41.4)	17	(58.6)	0.8	(0.4;1.3)	0.292			
Urban	140	49	(35.0)	91	(65.0)	1.3	(0.6;3.0)	0.516			
**Age**											
[30–40[ years	183	52	(28.4)	131	(71.6)	1			1		
[40–50[ years	117	47	(40.2)	70	(59.8)	1.7	(1.0;2.8)	**0.035**	2.1	(1.2;3.7)	**0.007**
**Matrimonial status**											
Single	47	13	(27.7)	34	(72.3)	1					
In a relationship	253	86	(34.0)	167	(66.0)	1.3	(0.7;2.7)	0.398			
**Level of instruction**											
No school/primary	94	29	(30.9)	65	(69.1)	1					
Secondary	153	52	(34.0)	101	(66.0)	1.1	(0.6;2.0)	0.610			
Higher	53	18	(34.0)	35	(66.0)	1.1	(0.6;2.4)	0.698			
**Religion**											
Monotheism	241	80	(33.2)	161	(66.8)	1					
Animist	36	12	(33.3)	24	(66.7)	1.0	(0.5;2.1)	0.987			
Atheist/others	23	7	(30.4)	16	(69.6)	0.9	(0.3;2.2)	0.788			
**Women’s monthly income**											
≤ 50 000 XAF	251	74	(29.5)	177	(70.5)	1			1		
> 50.000 XAF	46	22	(47.8)	24	(52.2)	2.2	(1.2;4.1)	**0.016**	2.2	(1.0;5.0)	**0.049**
**Household’s monthly income**											
≤ 100,000	213	63	(29.6)	150	(70.4)	1			1		
> 100,000	67	27	(40.3)	40	(59.7)	1.6	(0.9;2.8)	0.103	1.2	(0.6;2.5)	0.579
**CC knowledge level**											
Insufficient	112	25	(22.3)	87	(77.7)	1			1		
Adequate	188	74	(39.4)	114	(60.6)	2.2	(1.3;3.8)	**0.003**	2.6	(1.4;4.7)	**0.001**

CC: cervical cancer, cOR: crude odds ratio, 95%CI: 95% confidence interval, p: probability value, aOR: adjusted odds ratio

### Implementation preferences of home-based HPV self-sampling

**Fig. 4:**
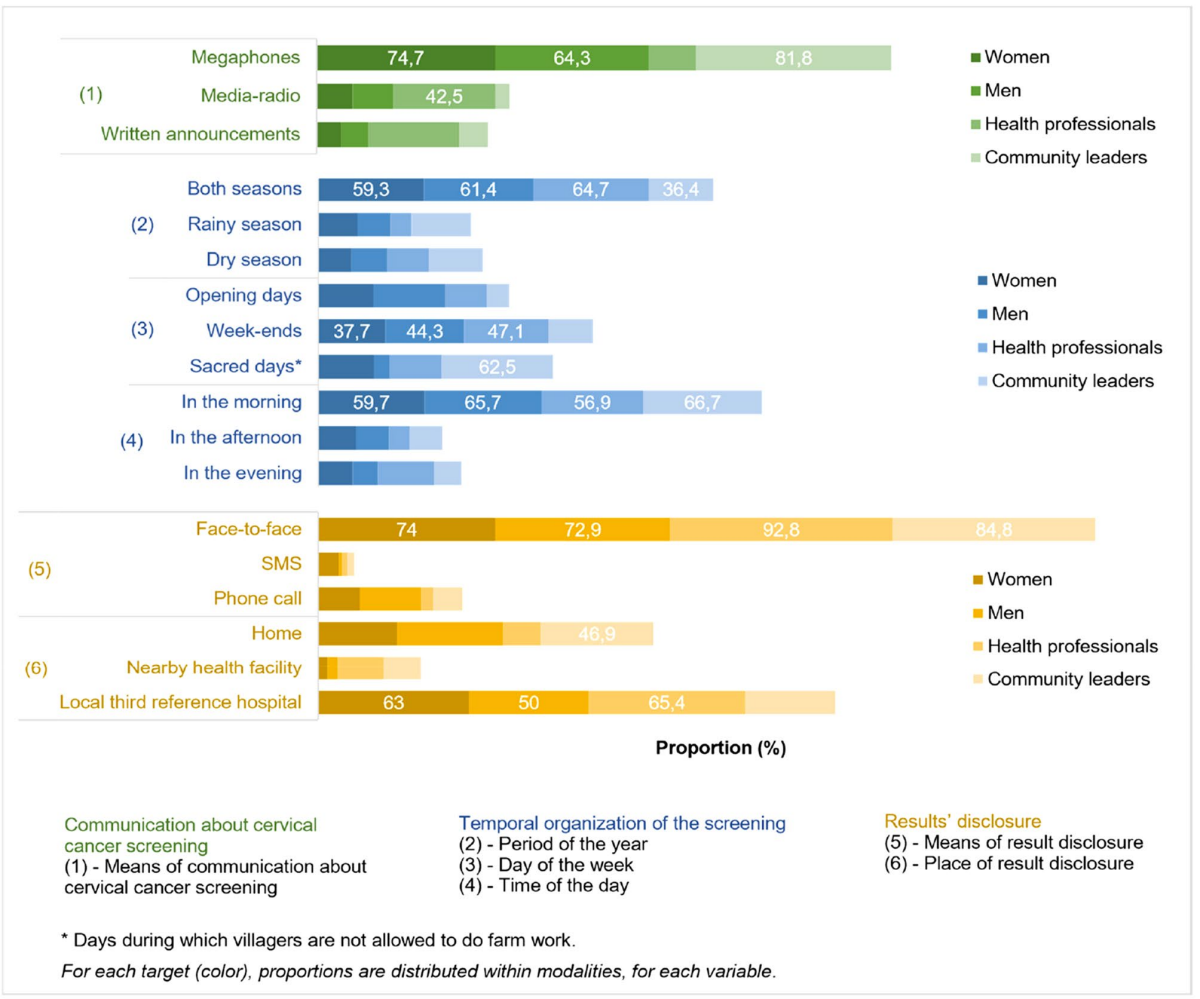
Implementation preferences of home-based HPV self-sampling

Given their relevance in the implementation of home-based HPV self-sampling, the perspectives of all four stakeholder groups were consulted to identify their preferred timing (season and time of day) and communication strategies (Fig. 4).

More than average of the women, men, and HCPs had no seasonal preference (59.3%, 61.4%, and 64.7%, respectively). In contrast, a majority of community leaders (69%) preferred that home-based HPV self-sampling be conducted during the rainy season. Among all stakeholder groups, morning periods were the preferred time for HPV self-sampling. A good number of community leaders (62.5%) emphasized on the importance of aligning the approach with culturally designated “sacred days” which are weekly rest days during which no farming or outdoor labour is carried out in the community.

When participants were asked about their preferred means of sensitization about home-based HPV self-sampling, women (70.0%), men (64.3%), and community leaders (60.6%), favoured the use of megaphones over radio or written announcements. HCPs were more inclined toward communication via the media (42.5%). Furthermore, participants clearly insisted that communication should not be made by community health workers alone. Women strongly adhered to this view, with 74.7% expressing disagreement with relying exclusively on community health workers.

Concerning the disclosure of HPV test results, a strong preference for face-to-face communication was observed among women (74.0%), men (72.9%), HCPs (92.8%), and community leaders (84.8%). Furthermore, the preferred location for receiving test results was the Dschang Regional Annex Hospital, as indicated by women (63.0%), men (50.0%), and HCPs (65.4%). But some community leaders preferred that results be disclosed at home (46.9%).

## Discussion

This study confirms the high acceptability of home-based HPV self-sampling in the West Region of Cameroon. While hospital-based screening is often limited by time, distance, and costs [9–14], home-based HPV self-sampling offers a promising alternative. Our findings suggest the need to address factors influencing screening practices as well as the role of men, community leaders and practical implementation aspects for its success.

### Socio demographic characteristics

Our study included 300 women, 70 men, 33 community leaders, and 153 healthcare professionals. Many lived in urban areas, were married, and worked in the informal sector. Only one quarter of women’s partners participated, mainly due to daily occupations, family responsibilities, or single status. Similar challenges in involving men have been observed in other African settings, often linked to economic pressures and limited availability [24–26].

Although home-based HPV self-sampling was well accepted, socioeconomic constraints continued to influence screening behaviours in all settings.

### Association between age, monthly income and practice of the screening

Most participants’ revenues are below 50,000 XAF (~ 80 euros), just slightly above the estimated minimum wage in Cameroon (41,875 XAF) [27]. Women with higher income were more likely to have been screened, because they face fewer barriers such as transport costs or children care. This aligns with the 2018 Cameroon Demographic and Health Survey, which showed an increase in CC screening rates (from 1 to 8%) with better economic conditions [28]. Higher income is linked with better health outcomes in LMIC [29–31]. These results highlight the impact of economic disparities and social determinants of health—such as income, transportation, and living conditions—on access to care, health behaviours, and outcomes [32–34]. They need to be well addressed by public health programs in order to enhance screening uptake. Home-based HPV self-sampling could alleviate some of the financial burden and hence improve access.

### Association between level of knowledge and practice of cervical cancer screening

Our study found a significant association between the knowledge of CC screening and its practice, highlighting knowledge as a crucial determinant of health behaviour [14, 15]. Overall, 65% of participants had good knowledge of CC—higher than the 46% reported in the 2018 Cameroon Demographic and Health Survey. Whereas, only 28% knew about screening and 4% had ever been screened [28]. However, there were some differences between the questionnaire used in this study and that in the health survey. This rate is also higher than that in South Africa (28%) [35], where there is a clear need to improve awareness. Although instruction level is generally linked with better CC knowledge, as seen in Saudi Arabia [36], our study did not find a direct association between education and screening. This is due to the relatively high level of instruction of our sample (over 60% with at least secondary school level). However, specific knowledge of CC was positively associated with screening. Thus, highlighting the value of targeted education to boost participation, as shown in other African contexts [37–39]. Therefore, enhancing knowledge among women, families, and community leaders is a key issue to improving screening uptake, regardless of the screening setting. Home-based HPV self-sampling could offer a promising avenue to improve knowledge, as healthcare professionals can directly address information gaps during home visits.

### Male partners and community leaders’ roles in cervical cancer screening

Men were included in this study because of their key role in family decision-making. Particularly in West African contexts where they act as heads of household [40]. While many supported home-based HPV self-sampling, one-third expressed negative attitudes toward CC screening. Similar findings in Uganda, Kenya, and Ethiopia link limited knowledge of CC risks, prevention and treatment to male reluctance. This is in line with previous research that highlighted men as both barriers to and supporters of CC screening initiatives [13, 41–44]. These findings suggest that enhancing male understanding could lead to greater support [42, 43, 45]. Yet, as the reasons for their negative attitudes were not fully explored in our study, further qualitative research would be required.

Community leaders also emerged as essential allies, with 90% expressing supportive views. Their influence on health behaviours is well documented in sub-Saharan Africa [46–49]. That underscores the importance of involving them at an early stage to CC screening initiatives.

### Acceptability of home-based HPV self-sampling

Home-based HPV self-sampling was well accepted by women in our study (73.7%). Therefore, supporting its potential to improve screening uptake and advance WHO’s elimination goals. Additionally, our study found that one out of three women had participated in CC screening 5 years ago, what aligns with national statistics (4%) [28]. This positive outreach is eventually due to the availability of a free screening program, implemented as a pilot project prior to the 3T study since 2016. However, as previous research on 3T shows, financial access alone does not eliminate structural and individual barriers [10]. Research from Australia and Spain has suggested that home-based self-sampling can reduce common barriers such as embarrassment, time constraints, and fear of pain [50, 51]. HPV self-sampling is highly acceptable for CC screening. It offers the possibility to reach many unscreened women and increase CC screening coverage in sub-Saharan Africa [52, 53]. In our study, most women and their relatives expressed confidence in women’s ability to perform self-sampling correctly. Thus, reinforcing its suitability for task-shifting strategies [17, 18].

Notably, in eight of the nine health areas included in the study, the acceptance of home-based HPV self-sampling was very high (Fig. 3). However, in one health area (Fontsa Touala) with poor accessibility (Fig. 2), the acceptance rate was only moderate (additional file 2). Specifically, 9 out of the 11 women recruited in this health area expressed concerns that their home environment was not clean or hygienic enough to perform the HPV self-sampling. Additionally, some participants mentioned the distance to the health facility as a factor that could discourage them from seeking screening at hospital. These challenges are similar to findings from other studies on geographic and infrastructure-related barriers to CC screening [10, 13, 15, 54, 55]. Home-based self-sampling can help to reduce these obstacles, especially in rural regions like West Cameroon, where poor roads and reliance on motorbikes limit hospital access (Fig. 2).

### Implementation perspectives of home-based HPV self-sampling

Effective communication between communities and HCPs is essential for successful CC screening programs, especially in low and middle income countries such as Cameroon. Community health workers play a key role in raising awareness, particularly in rural areas [56, 57]. Nevertheless, they should not be the sole communication channel, as they are not health experts. In Nigeria, Olubodun et al. recommend using diverse tools—SMS, phone calls, and town criers (individuals responsible for proclaiming official announcements in public, using tools such as bells, drums, or megaphones)—to enhance outreach [58]. Our findings corroborate this, showing a strong preference for megaphones as a means to reach large groups in public spaces (Fig. 4). This is consistent with Thoma’s observation that megaphones often outperform posters and media broadcasts [59].

In order to improve participation, screening activities should be in harmony with local routines. Participants favoured morning visits and HPV self-sampling on “sacred days”—culturally significant days when people remain at home. These community rest days provide strategic opportunities to reach more individuals while respecting local customs, similar to designated public holidays [60].

Although the HPV self-sampling was conducted at home, most participants preferred receiving their results at health facilities to avoid stigmatisation, especially in case of positive results [10]. This echoes findings from Gambia, where stigmatisation related to COVID-19 reduced acceptance of home-based follow-ups [61]. This underlines the importance of culturally sensitive communication, and the need for HCPs to be trained not only in clinical delivery but also in respectful, confidential result disclosure to maintain community harmony.

### Strengths and limitations

To our knowledge, this is the pioneer quantitative study in sub-Saharan Africa to explore the acceptability and preferences for implementing home-based HPV self-sampling. However, the study presents several limitations. Firstly, it was carried out before the actual rollout of home-based HPV self-sampling, as part of a pre-implementation phase. This means we could not assess how people behave in real conditions, but the findings provide useful guidance for planning future programs. Secondly, the cross-sectional design does not allow the establishment of causal relationships. Finally, fewer men and close relatives were recruited than expected. Which reflects common difficulties in involving male partners in previous African studies [24, 25].

Despite these limitations, the study has several strengths. It includes an extensive and diverse set of participants across four different groups, all without prior experience of home-based self-sampling. The high number of women involved, as well as the inclusion of healthcare professionals and community leaders, helped capture a wide range of perspectives. We also reached participants from various health areas, with different levels of accessibility, including urban, semi-urban, and rural communities. These aspects increase the relevance and potential use of our findings in similar contexts.

The upcoming CASAHO A study which will compare home and hospital-based screening, will further explore key operational aspects such as kit delivery, results communication, and follow-up.

## Conclusion

In our context, home-based HPV self-sampling was largely supported by stakeholders. This approach provides an important opportunity to reach women who have never participated in health facility-based CC screening. As was the case for 73.1% of women in our setting. However, community involvement, prior to the implementation of CC screening is crucial to ensure the acceptance of new screening programs with respect to awareness campaigns, timing of screening, and means of results disclosure.

## Supplementary Information

Below is the link to the electronic supplementary material.

Supplementary Material 1

Supplementary Material 2

Supplementary Material 3

Supplementary Material 4

## Data Availability

The datasets used and/or analysed during the current study are available from the corresponding author upon reasonable request. Due to privacy and ethical considerations, access to the data may be restricted to ensure confidentiality and compliance with institutional regulations.

## Footnotes

1 A religious leader guides spiritual and moral practices; his authority is based on religious beliefs. He influences individual and family values. A traditional leader preserves local customs and maintains social order. He often holds his powers from ancestors or family lineage. A social leader mobilises for societal causes and acts for change in the community. His influence derives from his charisma or public commitment.

## Abbreviations

CC: Cervical Cancer
DHD: Dschang Health District
HCPs: Healthcare professionals
HPV: Human papillomavirus
LMIC: Low-and-middle income country
WHO: World Health Organization
