# Supplementary material for: Acceptability of home-based HPV self-sampling for cervical cancer screening among users and providers in the West region of Cameroon: a cross-sectional study
Source: BMC Health Serv Res. 2025 Oct 3;25:1303. doi: 10.1186/s12913-025-13467-1 (PMC12495857; doi:10.1186/s12913-025-13467-1)
Supplement: Supplementary file 1 — Supplementary Material 1 [file 12913_2025_13467_MOESM1_ESM.docx]

**Additional file 1 : Questionnaires**

# **QUESTIONNAIRE « A » : DESTINE AUX FEMMES**

**ETUDE CASAHO**

**CERVICAL CANCER SCREENING AT HOME TO INCREASE PARTICIPATION: EXPLORING USERS’ AND PROVIDERS’ PERSPECTIVES**

**Introduction**

Bonjour Madame. Je me nomme DATCHOUA MOUKAM Alida, anthropologue et étudiante PhD en Santé Globale. Le but de mon travail est de mieux comprendre les motivations des femmes à participer à la prévention du cancer du col de l’utérus. Vos réponses à ce questionnaire sont importantes pour nous et nous permettrons d’identifier les barrières potentielles et éléments facilitateurs au dépistage du cancer du col de l’utérus à domicile.

Toutes vos réponses seront traitées de manière anonyme et vous pouvez à tout moment renoncer à participer à ce questionnaire.

Acceptez-vous de répondre à ce questionnaire ? Oui [__] Non [__]

| 1. **IDENTIFICATION DU QUESTIONNAIRE** | | | | | | |
| --- | --- | --- | --- | --- | --- | --- |
| **A1** | Date | ____/____/2023 | **A2** | Cible | | Femmes [FM] |
| **A3** | Enquêteur(trice) | \|___\|___\|___\| - \|___\| | **A4** | Code | | \| F \| M \| - \|___\| - \|___\| - \|___\|___\| |
|  |  | |  | | | |
| 1. **CARACTERISTIQUES SOCIODEMOGRAPHIQUES** | | | | | | |
| **B1** | Aire de santé | | [________________________] | | | |
| **B2** | Quel est votre âge ? | | [_____] ans | | | |
| **B3** | Quel est votre statut civil ? | | [_] Mariée [_] Célibataire [_] Divorcée [_] Veuve | | | |
| **B4** | Depuis combien de temps vivez-vous dans cette communauté ? | | [_] Moins d’un an [_] 1 an – 3 ans [_] 3 ans – 5 ans [_] 5 ans – 10 ans [_] > 10 ans [_] Toute ma vie | | | |
| **B5** | Quel est votre niveau d’étude ? | | [_] Primaire [_] Secondaire [_] Supérieur  [_] Non scolarisée | | | |
| **B6** | Quelle est votre profession ? | | [_] Secteur privé [_] Secteur public [_] Secteur informel  [_] Agricultrice [_] Ménagère [_] Sans emploi [_] Autre | | | |
| **B7** | Quel est le niveau d’étude de votre conjoint ? | | [_] Primaire [_] Secondaire [_] Supérieur  [_] Non scolarisée | | | |
| **B8** | Quelle est la profession de votre conjoint ? | | [_] Secteur privé [_] Secteur public [_] Secteur informel  [_] Agriculteur [_] Sans emploi  [_] Autre (_______________________) | | | |
| **B9** | Quelle est votre religion ? | | [_] Christianisme [_] Islam [_] Animisme  [_] Autre (_________) | | | |
| **B10** | Quel est votre revenu mensuel (en FCFA) ? | | [_] < 50.000 [_] 50.001 – 100.000 [_] 100.001 – 200.000 [_] > 200.000 | | | |
| **B11** | Quel est le revenu mensuel de votre ménage (en FCFA) ? | | [_] < 50.000 [_] 50.001 – 100.000 [_] 100.001 – 200.000 [_] > 200.000 | | | |
| **B12** | Combien d’enfants vivants avez-vous ? | | [_____] enfants | | | |
| **B13** | Combien d’enfants y a-t-il dans le ménage ? | | [_____] enfants | | | |
|  |  | |  | | | |
| 1. **CONNAISSANCES SUR LE CANCER DU COL DE L’UTERUS** | | | | | | |
| **C1** | Avez-vous déjà entendu parler du cancer du col de l’utérus ? | | [_] Oui [_] Non | | | |
|  | *Si « C1 = Oui », continuer le remplissage à partir de la question C2*  *Si « C1 = Non », passer à la rubrique « D - Dépistage du cancer du col de l’utérus et motivations »* | | | | | |
| **C2** | Quel est l’agent responsable du cancer du col de l’utérus ? | | [_] HPV (Human Papilloma Virus)  [_] VIH (Virus de l’Immunodéficience Humaine)  [_] Autre(s) (________________________________________________) | | | |
| **C3** | Quels sont les facteurs de risque favorisant une infection au HPV ? | | [_] Précocité des rapports sexuels  [_] Rapports sexuels non protégés  [_] Multiplicité des partenaires sexuels  [_] Multiparité  [_] Échange des objets souillés  [_] Autre(s) (________________________________________________) | | | |
| **C4** | Quelles sont les personnes les plus à risque de développer un cancer du col de l’utérus ? | | [_] Les personnes infectées par le VIH  [_] Les personnes immunodéprimées  [_] Les personnes qui fument  [_] Autre(s) (_______________________________________) | | | |
| **C5** | Le cancer du col de l’utérus peut-il se prévenir ? | | [_] Oui [_] Non | | | |
|  | *Si « C5 = Oui », continuer le remplissage à partir la question C6*  *Si « C5 = Non », passer à la question C7* | | | | | |
| **C6** | Comment peut se prévenir le cancer du col de l’utérus ? | | [_] Dépistage  [_] Vaccination contre le HPV  [_] Protection lors des rapports sexuels  [_] Rapports sexuels avec un nombre limité de partenaires  [_] Autre(s) (_______________________________________) | | | |
| **C7** | Le cancer du col de l’utérus peut-il se guérir ? | | [_] Oui [_] Non | | | |
| **C8** | Selon vous, auriez-vous plus peur de souffrir du cancer du col de l’utérus que d’autres maladies comme le paludisme ou le diabète ou l’hypertension ? | | [_] Parfaitement en désaccord  [_] En désaccord  [_] Neutre  [_] En accord  [_] Parfaitement en accord | | | |
|  |  | |  | | | |
| 1. **DEPISTAGE DU CANCER DU COL DE L’UTERUS ET MOTIVATIONS** | | | | | | |
| **D1** | Avez-vous déjà bénéficié d’un dépistage du cancer du col de l’utérus ?  Si **Oui**, quand ? | | [_] Oui [_] Non    *[_] < 5 ans [_] < 5 ans* | | | |
|  | *Si « D1 = Oui », passer à la question D3 et continuer le remplissage plus bas*  *Si « D1 = Non », répondre à la question D2, sauter la question D3 et continuer le remplissage à partir de la question D4* | | | | | |
| **D2** | Pour quelles raisons n’avez-vous pas fait de dépistage ? | | [_] Manque de temps [_] Manque de volonté  [_] Maladie mystique . [_] Peur de la douleur  [_] Manque d’information sur le dépistage  [_] Peur d’être contaminée à l’hôpital  [_] Indisponibilité du dépistage  [_] Dépistage coûteux  [_] Autres (__________________________________) | | | |
| **D3** | Qu’est-ce qui vous a motivée à faire ce dépistage ? | | [_] Vous êtes soucieuse de votre santé  [_] Vous ressentez des symptômes gynécologiques  [_] Antécédents de cancer du col dans l’entourage  [_] Vous avez été convaincue par votre entourage  [_] Vous avez été sensibilisée par un agent de santé ou un.e infirmier.ère  [_] Autre(s) (_______________________________________________ ________________________________________________ ________________________________________________) | | | |
|  | **Comment vous sentez-vous ou appréciez-vous les éléments suivants, relativement au dépistage du cancer du col de l’utérus à la maison ?** | | | | | |
| **D4** | Je serais embarrassée de faire l’auto-prélèvement à la maison | | | | [_] Pas du tout d’accord [_] Pas d’accord  [_] Neutre  [_] D’accord [_] Tout à fait d’accord | |
| **D5** | J’ai peur de ne pas faire le prélèvement correctement à la maison | | | | [_] Pas du tout d’accord [_] Pas d’accord  [_] Neutre  [_] D’accord [_] Tout à fait d’accord | |
| **D6** | J’ai peur de ce que les autres à la maison pourraient penser de mon statut/résultat | | | | [_] Pas du tout d’accord [_] Pas d’accord  [_] Neutre  [_] D’accord [_] Tout à fait d’accord | |
| **D7** | J’ai peur de mettre les autres à la maison mal à l’aise | | | | [_] Pas du tout d’accord [_] Pas d’accord  [_] Neutre  [_] D’accord [_] Tout à fait d’accord | |
| **D8** | Je suis à l’aise avec le fait de recevoir une éducation sur le cancer du col de l’utérus à la maison | | | | [_] Pas du tout d’accord [_] Pas d’accord  [_] Neutre  [_] D’accord [_] Tout à fait d’accord | |
| **D9** | La distance entre la maison et l’hôpital le plus proche offrant le dépistage du cancer du col de l’utérus est longue et ne m’encourage pas à m’y rendre pour faire le dépistage | | | | [_] Pas du tout d’accord [_] Pas d’accord  [_] Neutre  [_] D’accord [_] Tout à fait d’accord | |
| **D10** | La rapidité d’exécution du dépistage m’encourage à le faire | | | | [_] Pas du tout d’accord [_] Pas d’accord  [_] Neutre  [_] D’accord [_] Tout à fait d’accord | |
| **D11** | La maison est suffisamment saine et hygiénique pour y faire le dépistage | | | | [_] Pas du tout d’accord [_] Pas d’accord  [_] Neutre  [_] D’accord [_] Tout à fait d’accord | |
| **D12** | S’il le sait, mon partenaire peut me décourager ou m’empêcher de faire le dépistage à la maison | | | | [_] Pas du tout d’accord [_] Pas d’accord  [_] Neutre  [_] D’accord [_] Tout à fait d’accord | |
| **D13** | Les autres membres de la maison peuvent me décourager ou m’empêcher de faire le dépistage à la maison | | | | [_] Pas du tout d’accord [_] Pas d’accord  [_] Neutre  [_] D’accord [_] Tout à fait d’accord | |
| **D14** | Où préfèreriez-vous réaliser votre dépistage du cancer du col de l’utérus ? | | [_] A l’hôpital [_] Au centre de santé [_] A la maison  [_] Autre (________________________)  [_] Ne sait pas  [_] Ne souhaite jamais faire le dépistage | | | |
|  | *Si « D14 = Hôpital OU Centre de santé OU Maison OU Autre », répondre à la question D15*  *Si « D14 = Ne souhaite jamais faire le dépistage », répondre à la question D16* | | | | | |
| **D15** | Pourquoi préférez-vous ce lieu (plusieurs réponses possibles) ? | | [_] Je n’ai pas besoin de me déplacer pour l’hôpital  [_] Ça ne me coûte rien en temps et en argent  [_] Je n’aurai pas besoin de patienter avant d’être prise en charge  [_] La discrétion pourra être mieux respectée à la maison  [_] Je me sens plus à l’aise de faire le prélèvement chez moi  [_] Le test pourrait ne pas être bien fait à la maison  [_] La maison n’est pas très propre  [_] Mon partenaire ne sera pas d’accord avec le test à la maison  [_] Je me sens plus à l’aise de faire le prélèvement à l’hôpital  [_] On ne sait jamais, quelque chose d’imprévu peut se passer  [_] L’accueil n’est pas agréable dans l’hôpital  [_] Autre(s)  (________________________________________________ _________________________________________________ _________________________________________________) | | | |
| **D16** | Pourquoi ne souhaitez-vous jamais faire le dépistage ? | | [_] Ça ne m’intéresse pas  [_] Personne dans mon entourage n’a (n’a eu) ça  [_] J’ai peur du résultat  [_] Autre(s)  (_______________________________________________ ________________________________________________ ________________________________________________) | | | |
|  |  | |  | | | |
| 1. **FAISABILITE DU DEPISTAGE SELON LES FEMMES** | | | | | | |
|  | **Afin d’optimiser la mise en œuvre du dépistage à domicile, selon vous :** | | | | | |
| **E1** | Pensez-vous que l’auto-prélèvement à domicile puisse être proposé en communauté par les ARC seuls ? | | [_] Pas du tout d’accord  [_] Pas d’accord  [_] Neutre  [_] D’accord  [_] Tout à fait d’accord | | | |
| **E2** | Quelle période de l’année est plus indiquée pour proposer le dépistage à domicile ? | | [_] Saison des pluies [_] Saison sèche [_] Autant la saison sèche que la saison des pluies | | | |
| **E3** | Quels jours de la semaine sont plus indiqués pour proposer le dépistage à domicile ? | | [_] Les jours ouvrables [_] Les week-ends [_] Les jours sacrés | | | |
| **E4** | Quel moment de la journée est plus indiqué pour proposer le dépistage à domicile ? | | [_] Le matin [_] Dans l’après-midi [_] La soirée | | | |
| **E5** | Quel(s) moyen(s), pour la communication des résultats aux femmes, vous est (sont) indiqué(s) ? | | [_] Appel [_] Message [_] Face à face | | | |
| **E6** | Quel(s) lieu(x), pour la communication des résultats, vous semble(nt) indiqué(s) ? | | [_] Hôpital Régional Annexe [_] Maison [_] Centre de santé du village | | | |
| **E7** | Quel(s) moyen(s) vous semble(nt) adapté(s) pour la communication sur le dépistage à domicile ? | | [_] Média-radio [_] Communiqués écrits [_] Mégaphones via ARC [_] Autre(s) | | | |

**Merci pour votre participation**

# **QUESTIONNAIRE « B » : DESTINE AUX HOMMES OU PROCHES PARENTS**

**ETUDE CASAHO**

**CERVICAL CANCER SCREENING AT HOME TO INCREASE PARTICIPATION: EXPLORING USERS’ AND PROVIDERS’ PERSPECTIVES**

**Introduction**

Bonjour Monsieur/Madame. Je me nomme DATCHOUA MOUKAM Alida, anthropologue et étudiante PhD en Santé Globale. Le but de mon travail est de mieux comprendre les motivations des femmes à participer à la prévention du cancer du col de l’utérus. Vos réponses à ce questionnaire sont importantes pour nous et nous permettrons d’identifier les barrières potentielles et éléments facilitateurs au dépistage du cancer du col de l’utérus à domicile.

Toutes vos réponses seront traitées de manière anonyme et vous pouvez à tout moment renoncer à participer à ce questionnaire.

Acceptez-vous de répondre à ce questionnaire ? Oui [__] Non [__]

| 1. **IDENTIFICATION DU QUESTIONNAIRE** | | | | | | |
| --- | --- | --- | --- | --- | --- | --- |
| **A1** | Date | ____/____/2023 | **A2** | | Cible | Hommes [HM] |
| **A3** | Enquêteur(trice) | \|___\|___\|___\| - \|___\| | **A4** | | Code | \| H \| M \| - \|___\| - \|___\| - \|___\|___\| |
|  |  | |  | | | |
| 1. **CARACTERISTIQUES SOCIODEMOGRAPHIQUES** | | | | | | |
| **B1** | Aire de santé | | [________________________] | | | |
| **B2** | Quel est votre âge ? | | [_____] ans | | | |
| **B3** | Quel est votre statut civil ? | | [_] Marié [_] Célibataire [_] Divorcé [_] Veuve | | | |
| **B4** | Depuis combien de temps vivez-vous dans cette communauté ? | | [_] Moins d’un an [_] 1 an – 3 ans [_] 3 ans – 5 ans [_] 5 ans – 10 ans [_] > 10 ans [_] Toute ma vie | | | |
| **B5** | Quel est votre niveau d’étude ? | | [_] Primaire [_] Secondaire [_] Supérieur  [_] Non scolarisé | | | |
| **B6** | Quelle est votre profession ? | | [_] Secteur privé [_] Secteur public [_] Secteur informel  [_] Agriculteur [_] Sans emploi [_] Autre | | | |
| **B7** | Quelle est votre religion ? | | [_] Christianisme [_] Islam [_] Animisme  [_] Autre (_________) | | | |
| **B8** | Quel est votre revenu mensuel (en FCFA) ? | | [_] < 50.000 [_] 50.001 – 100.000 [_] 100.001 – 200.000 [_] > 200.000 | | | |
| **B9** | Quel est le revenu mensuel de votre ménage (en FCFA) ? | | [_] < 50.000 [_] 50.001 – 100.000 [_] 100.001 – 200.000 [_] > 200.000 | | | |
| **B10** | Combien d’enfants vivants avez-vous ? | | [_____] enfants | | | |
| **B11** | Combien d’enfants y a-t-il dans le ménage ? | | [_____] enfants | | | |
|  |  | |  | | | |
| 1. **CONNAISSANCES SUR LE CANCER DU COL DE L’UTERUS** | | | | | | |
| **C1** | Avez-vous déjà entendu parler du cancer du col de l’utérus ? | | [_] Oui [_] Non | | | |
|  | *Si « C1 = Oui », continuer le remplissage à partir de la question C2*  *Si « C1 = Non », passer à la rubrique « D - Dépistage du cancer du col de l’utérus et motivations »* | | | | | |
| **C2** | Quel est l’agent responsable du cancer du col de l’utérus ? | | [_] HPV (Human Papilloma Virus)  [_] VIH (Virus de l’Immunodéficience Humaine)  [_] Autre(s) (________________________________________________) | | | |
| **C3** | Quels sont les facteurs de risque favorisant une infection au HPV ? | | [_] Précocité des rapports sexuels  [_] Rapports sexuels non protégés  [_] Multiplicité des partenaires sexuels  [_] Multiparité  [_] Échange des objets souillés  [_] Autre(s) (________________________________________________) | | | |
| **C4** | Quelles sont les personnes les plus à risque de développer un cancer du col de l’utérus ? | | [_] Les personnes infectées par le VIH  [_] Les personnes immunodéprimées  [_] Les personnes qui fument  [_] Autre(s) (________________________________________________) | | | |
| **C5** | Le cancer du col de l’utérus peut-il se prévenir ? | | [_] Oui [_] Non | | | |
|  | *Si « C5 = Oui », continuer le remplissage à partir la question C6*  *Si « C5 = Non », passer à la question C7* | | | | | |
| **C6** | Comment peut se prévenir le cancer du col de l’utérus ? | | [_] Dépistage  [_] Vaccination contre le HPV  [_] Protection lors des rapports sexuels  [_] Rapports sexuels avec un nombre limité de partenaires  [_] Autre (_______________________________) | | | |
| **C7** | Le cancer du col de l’utérus peut-il se guérir ? | | [_] Oui [_] Non | | | |
| **C8** | Selon vous, auriez-vous plus peur que votre femme souffre du cancer du col de l’utérus que d’autres maladies comme le paludisme ou le diabète ou l’hypertension ? | | [_] Parfaitement en désaccord  [_] En désaccord  [_] Neutre  [_] En accord  [_] Parfaitement en accord | | | |
|  |  | |  | | | |
| 1. **DEPISTAGE DU CANCER DU COL DE L’UTERUS ET MOTIVATIONS** | | | | | | |
|  | **Comment vous sentez-vous ou appréciez-vous les éléments suivants, relativement au dépistage du cancer du col de l’utérus à la maison par votre femme ?** | | | | | |
| **D1** | Je serais embarrassée que ma femme fasse l’auto-prélèvement à la maison | | | [_] Pas du tout d’accord  [_] Pas d’accord  [_] Neutre  [_] D’accord  [_] Tout à fait d’accord | | |
| **D2** | J’ai peur que ma femme ne fasse pas correctement le prélèvement à la maison | | | [_] Pas du tout d’accord  [_] Pas d’accord  [_] Neutre  [_] D’accord  [_] Tout à fait d’accord | | |
| **D3** | J’ai peur de ce que les autres à la maison pourraient penser du statut/résultat de ma femme | | | [_] Pas du tout d’accord  [_] Pas d’accord  [_] Neutre  [_] D’accord  [_] Tout à fait d’accord | | |
| **D4** | J’ai peur qu’elle mette les autres à la maison mal à l’aise | | | [_] Pas du tout d’accord  [_] Pas d’accord  [_] Neutre  [_] D’accord  [_] Tout à fait d’accord | | |
| **D5** | Je suis à l’aise avec le fait que ma femme reçoive une éducation sur le cancer du col de l’utérus à la maison | | | [_] Pas du tout d’accord  [_] Pas d’accord  [_] Neutre  [_] D’accord  [_] Tout à fait d’accord | | |
| **D6** | La distance entre la maison et l’hôpital le plus proche offrant le dépistage du cancer du col de l’utérus est longue et n’encourage pas ma femme à s’y rendre pour faire le dépistage | | | [_] Pas du tout d’accord  [_] Pas d’accord  [_] Neutre  [_] D’accord  [_] Tout à fait d’accord | | |
| **D7** | La rapidité d’exécution du dépistage m’encourage à encourager ma femme à le faire | | | [_] Pas du tout d’accord  [_] Pas d’accord  [_] Neutre  [_] D’accord  [_] Tout à fait d’accord | | |
| **D8** | La maison est suffisamment saine et hygiénique pour y faire le dépistage | | | [_] Pas du tout d’accord  [_] Pas d’accord  [_] Neutre  [_] D’accord  [_] Tout à fait d’accord | | |
| **D9** | Les autres membres de la maison peuvent décourager ou empêcher ma femme de faire le dépistage à la maison | | | [_] Pas du tout d’accord  [_] Pas d’accord  [_] Neutre  [_] D’accord  [_] Tout à fait d’accord | | |
| **D10** | Seriez-vous d’accord que votre femme fasse son prélèvement à la maison ? | | [_] Oui [_] Non | | | |
| **D11** | Pourquoi cet avis ? | | [_] Ça ne me coûte rien [_] C’est pour la santé  [_] Je me soucie de ma femme [_] C’est sur place  [_] C’est rapide [_] Ça ne sert à rien  [_] C’est une conspiration [_] Elle n’est pas malade  [_] Je serais gêné [_] L’entourage pourrait être gêné [_] Autre(s)  (__________________________________________________________________________________________________) | | | |
|  |  | |  | | | |
| 1. **FAISABILITE DU DEPISTAGE SELON LES HOMMES / PATERNAIRES / PROCHES PARENTS** | | | | | | |
| **E1** | Pensez-vous que l’auto-prélèvement à domicile puisse être proposé en communauté par les ARC seuls ? | | [_] Pas du tout d’accord  [_] Pas d’accord  [_] Neutre  [_] D’accord  [_] Tout à fait d’accord | | | |
|  | **Afin d’optimiser la mise en œuvre du dépistage à domicile, selon vous :** | | | | | |
| **E2** | Quelle période de l’année est plus indiquée pour proposer le dépistage à domicile ? | | [_] Saison des pluies [_] Saison sèche [_] Autant la saison sèche que la saison des pluies | | | |
| **E3** | Quels jours de la semaine sont plus indiqués pour proposer le dépistage à domicile ? | | [_] Les jours ouvrables [_] Les week-ends [_] Les jours sacrés | | | |
| **E4** | Quel moment de la journée est plus indiqué pour proposer le dépistage à domicile ? | | [_] Le matin [_] Dans l’après-midi [_] La soirée | | | |
| **E5** | Quel(s) moyen(s), pour la communication des résultats aux femmes, vous semble(nt) indiqué(s) ? | | [_] Appel [_] Message [_] Face à face | | | |
| **E6** | Quel(s) lieu(x), pour la communication des résultats, vous semble(nt) indiqué(s) ? | | [_] Hôpital Régional Annexe [_] Maison [_] Centre de santé du village | | | |
| **E7** | Quel(s) moyen(s) vous semble(nt) adapté(s) pour la communication sur le dépistage à domicile ? | | [_] Média-radio [_] Communiqués écrits [_] Mégaphones via ARC [_] Autre(s) | | | |

**Merci pour votre participation**

**QUESTIONNAIRE « C » : DESTINE AUX LEADERS COMMUNAUTAIRES ET AUX MATRONES**

**ETUDE CASAHO**

**CERVICAL CANCER SCREENING AT HOME TO INCREASE PARTICIPATION: EXPLORING USERS’ AND PROVIDERS’ PERSPECTIVES**

**Introduction**

Bonjour Madame. Je me nomme DATCHOUA MOUKAM Alida, anthropologue et étudiante PhD en Santé Globale. Le but de mon travail est de mieux comprendre les motivations des femmes à participer à la prévention du cancer du col de l’utérus. Vos réponses à ce questionnaire sont importantes pour nous et nous permettrons d’identifier les barrières potentielles et éléments facilitateurs au dépistage du cancer du col de l’utérus à domicile.

Toutes vos réponses seront traitées de manière anonyme et vous pouvez à tout moment renoncer à participer à ce questionnaire.

Acceptez-vous de répondre à ce questionnaire ? Oui [__] Non [__]

| 1. **IDENTIFICATION DU QUESTIONNAIRE** | | | | | | |
| --- | --- | --- | --- | --- | --- | --- |
| **A1** | Date | ____/____/2023 | **A2** | | Cible | Leaders communautaires [LC] |
| **A3** | Enquêteur(trice) | \|___\|___\|___\| - \|___\| | **A4** | | Code | \| L \| C \| - \|___\| - \|___\| - \|___\|___\| |
|  |  | |  | | | |
| 1. **CARACTERISTIQUES SOCIODEMOGRAPHIQUES** | | | | | | |
| **B1** | Aire de santé | | [________________________] | | | |
| **B2** | Quel est votre âge ? | | [_____] ans | | | |
| **B3** | Quel est votre genre ? | | [_] Homme [_] Femme | | | |
| **B4** | Quel est votre statut civil ? | | [_] Marié(e) [_] Célibataire [_] Divorcé(e) [_] Veuve | | | |
| **B5** | Depuis combien de temps vivez-vous dans cette communauté ? | | [_] Moins d’un an [_] 1 an – 3 ans [_] 3 ans – 5 ans [_] 5 ans – 10 ans [_] > 10 ans [_] Toute ma vie | | | |
| **B6** | Quel est votre niveau d’étude ? | | [_] Primaire [_] Secondaire [_] Supérieur  [_] Non scolarisé(e) | | | |
| **B7** | Quel est votre rôle dans la communauté ? | | [_] Chef de quartier [_] Chef de village [_] Notable  [_] Patriarche/Matriarche [_] Leader religieux [_] Matrone  [_] Autre (_________) | | | |
| **B8** | Quelle est votre religion ? | | [_] Christianisme [_] Islam [_] Animisme  [_] Autre (_________) | | | |
|  |  | |  | | | |
| 1. **CONNAISSANCES SUR LE CANCER DU COL DE L’UTERUS** | | | | | | |
| **C1** | Avez-vous déjà entendu parler du cancer du col de l’utérus ? | | [_] Oui [_] Non | | | |
|  | *Si « C1 = Oui », continuer le remplissage à partir de la question C2*  *Si « C1 = Non », passer à la rubrique « D - Faisabilité du dépistage selon les leaders communautaires »* | | | | | |
| **C2** | Quel est l’agent responsable du cancer du col de l’utérus ? | | [_] HPV (Human Papilloma Virus)  [_] VIH (Virus de l’Immunodéficience Humaine)  [_] Autre(s) (________________________________________________) | | | |
| **C3** | Quels sont les facteurs de risque favorisant une infection au HPV ? | | [_] Précocité des rapports sexuels  [_] Rapports sexuels non protégés  [_] Multiplicité des partenaires sexuels  [_] Multiparité  [_] Échange des objets souillés  [_] Autre(s) (________________________________________________) | | | |
| **C4** | Quelles sont les personnes les plus à risque de développer un cancer du col de l’utérus ? | | [_] Les personnes infectées par le VIH  [_] Les personnes immunodéprimées  [_] Les personnes qui fument  [_] Autre(s) (________________________________________________) | | | |
| **C5** | Le cancer du col de l’utérus peut-il se prévenir ? | | [_] Oui [_] Non | | | |
|  | *Si « C5 = Oui », continuer le remplissage à partir la question C6*  *Si « C5 = Non », passer à la question C7* | | | | | |
| **C6** | Comment peut se prévenir le cancer du col de l’utérus (plusieurs réponses possibles) ? | | [_] Dépistage  [_] Vaccination contre le HPV  [_] Protection lors des rapports sexuels  [_] Rapports sexuels avec un nombre limité de partenaires  [_] Autre (_______________________________) | | | |
| **C7** | Le cancer du col de l’utérus peut-il se guérir ? | | [_] Oui [_] Non | | | |
| **C8** | Selon vous, auriez-vous plus peur qu’une femme souffre du cancer du col de l’utérus que d’autres maladies comme le paludisme ou le diabète ou l’hypertension ? | | [_] Pas du tout d’accord  [_] Pas d’accord  [_] Neutre  [_] D’accord  [_] Tout à fait d’accord | | | |
|  |  | |  | | | |
| **D. DEPISTAGE DU CANCER DU COL DE L’UTERUS ET MOTIVATIONS** | | | | | | |
|  | **Comment vous sentez-vous ou appréciez-vous les éléments suivants, relativement au dépistage du cancer du col de l’utérus à la maison par une femme de votre communauté ?** | | | | | |
| **D1** | Je serais embarrassée qu’une femme de ma communauté fasse l’auto-prélèvement à la maison | | | [_] Pas du tout d’accord  [_] Pas d’accord  [_] Neutre  [_] D’accord  [_] Tout à fait d’accord | | |
| **D2** | J’ai peur qu’une femme de ma communauté ne fasse pas correctement le prélèvement à la maison | | | [_] Pas du tout d’accord  [_] Pas d’accord  [_] Neutre  [_] D’accord  [_] Tout à fait d’accord | | |
| **D3** | J’ai peur de ce que les autres à la maison pourraient penser du statut/résultat de cette femme | | | [_] Pas du tout d’accord  [_] Pas d’accord  [_] Neutre  [_] D’accord  [_] Tout à fait d’accord | | |
| **D4** | J’ai peur qu’elle mette les autres à la maison mal à l’aise | | | [_] Pas du tout d’accord  [_] Pas d’accord  [_] Neutre  [_] D’accord  [_] Tout à fait d’accord | | |
| **D5** | Je suis à l’aise avec le fait qu’une femme de ma communauté reçoive une éducation sur le cancer du col de l’utérus à la maison | | | [_] Pas du tout d’accord  [_] Pas d’accord  [_] Neutre  [_] D’accord  [_] Tout à fait d’accord | | |
| **D6** | La distance entre la maison et l’hôpital le plus proche offrant le dépistage du cancer du col de l’utérus est longue et n’encourage pas les femmes de ma communauté à s’y rendre pour faire le dépistage | | | [_] Pas du tout d’accord  [_] Pas d’accord  [_] Neutre  [_] D’accord  [_] Tout à fait d’accord | | |
| **D7** | La rapidité d’exécution du dépistage m’encourage à encourager les femmes de ma communauté à le faire | | | [_] Pas du tout d’accord  [_] Pas d’accord  [_] Neutre  [_] D’accord  [_] Tout à fait d’accord | | |
| **D8** | La maison est suffisamment saine et hygiénique pour y faire le dépistage | | | [_] Pas du tout d’accord  [_] Pas d’accord  [_] Neutre  [_] D’accord  [_] Tout à fait d’accord | | |
| **D9** | Les autres membres de la maison peuvent décourager ou empêcher la femme de ma communauté de faire le dépistage à la maison | | | [_] Pas du tout d’accord  [_] Pas d’accord  [_] Neutre  [_] D’accord  [_] Tout à fait d’accord | | |
| **D10** | Seriez-vous d’accord qu’une femme de votre communauté fasse son prélèvement à la maison ? | | [_] Oui [_] Non | | | |
| **D11** | Pourquoi cet avis ? | | [_] Ça ne me coûte rien [_] C’est pour la santé  [_] Je me soucie de ma femme [_] C’est sur place  [_] C’est rapide [_] Ça ne sert à rien  [_] C’est une conspiration [_] Elle n’est pas malade  [_] Je serais gêné [_] L’entourage pourrait être gêné [_] Autre(s)  (__________________________________________________________________________________________________) | | | |
|  |  | |  | | | |
| **E.** | **FAISABILITE DU DEPISTAGE SELON LES LEADERS COMMUNAUTAIRES** | | | | | |
|  | **Afin d’optimiser la mise en œuvre du dépistage à domicile, selon vous :** | | | | | |
| **E1** | Pensez-vous que l’auto-prélèvement à domicile puisse être proposé en communauté par les ARC seuls ? | | [_] Pas du tout d’accord  [_] Pas d’accord  [_] Neutre  [_] D’accord  [_] Tout à fait d’accord | | | |
| **E2** | Quelle période de l’année est plus indiquée pour proposer le dépistage à domicile ? | | [_] Saison des pluies [_] Saison sèche [_] Autant la saison sèche que la saison des pluies | | | |
| **E3** | Quels jours de la semaine sont plus indiqués pour proposer le dépistage à domicile ? | | [_] Les jours ouvrables [_] Les week-ends [_] Les jours sacrés | | | |
| **E4** | Quel moment de la journée est plus indiqué pour proposer le dépistage à domicile ? | | [_] Le matin [_] Dans l’après-midi [_] La soirée | | | |
| **E5** | Quel(s) moyen(s), pour la communication des résultats aux femmes, vous semble(nt) indiqué(s) ? | | [_] Appel [_] Message [_] Face à face | | | |
| **E6** | Quel(s) lieu(x), pour la communication des résultats, vous semble(nt) indiqué(s) ? | | [_] Hôpital Régional Annexe [_] Maison [_] Centre de santé du village | | | |
| **E7** | Quel(s) moyen(s) vous semble(nt) adapté(s) pour la communication sur le dépistage à domicile ? | | [_] Média-radio [_] Communiqués écrits [_] Mégaphones via ARC [_] Autre(s) | | | |

**Merci pour votre participation**

# **QUESTIONNAIRE « D » : DESTINE AUX PROFESSIONNELS DE SANTE**

**ETUDE CASAHO**

**CERVICAL CANCER SCREENING AT HOME TO INCREASE PARTICIPATION: EXPLORING USERS’ AND PROVIDERS’ PERSPECTIVES**

**Introduction**

Bonjour Madame. Je me nomme DATCHOUA MOUKAM Alida, anthropologue et étudiante PhD en Santé Globale. Le but de mon travail est de mieux comprendre les motivations des femmes à participer à la prévention du cancer du col de l’utérus. Vos réponses à ce questionnaire sont importantes pour nous et nous permettrons d’identifier les barrières potentielles et éléments facilitateurs au dépistage du cancer du col de l’utérus à domicile.

Toutes vos réponses seront traitées de manière anonyme et vous pouvez à tout moment renoncer à participer à ce questionnaire.

Acceptez-vous de répondre à ce questionnaire ? Oui [__] Non [__]

| 1. **IDENTIFICATION DU QUESTIONNAIRE** | | | | | | |
| --- | --- | --- | --- | --- | --- | --- |
| **A1** | Date | ____/____/2023 | **A2** | Cible | | Professionnels de santé [PS] |
| **A3** | Enquêteur(trice) | \|___\|___\|___\| - \|___\| | **A4** | Code | | \| P \| S \| - \|___\| - \|___\| - \|___\|___\| |
|  |  | |  | | | |
| 1. **CARACTERISTIQUES SOCIODEMOGRAPHIQUES** | | | | | | |
| **B1** | Aire de santé | | [________________________] | | | |
| **B2** | Quel est votre âge ? | | [____] ans | | | |
| **B3** | Quel est votre genre ? | | [_] Homme [_] Femme | | | |
| **B4** | Quel est votre statut civil ? | | [_] Marié(e) [_] Célibataire [_] Divorcé(e) [_] Veuve | | | |
| **B5** | Depuis combien de temps vivez-vous dans cette communauté ? | | [_] Moins d’un an [_] 1 an – 3 ans [_] 3 ans – 5 ans [_] 5 ans – 10 ans [_] > 10 ans [_] Toute ma vie | | | |
| **B6** | Quel est votre niveau d’étude ? | | [_] Primaire [_] Secondaire [_] Supérieur  [_] Non scolarisé(e) | | | |
| **B7** | Quelle est votre qualification ? | | [_] Aide-soignante [_] Infirmière [_] Sage-femme [_] Médecin [_] TMS [_] Autres (________) | | | |
| **B8** | Combien d’années d’ancienneté avez-vous ? | | [_] 1 an [_] ]1 – 3] ans [_] ]3 – 5] ans [_] >5 ans | | | |
| **B9** | Quel est le niveau d’étude de votre conjoint ? | | [_] Primaire [_] Secondaire [_] Supérieur  [_] Non scolarisée | | | |
| **B10** | Quelle est la profession de votre conjoint ? | | [_] Secteur privé [_] Secteur public [_] Secteur informel  [_] Agriculteur [_] Sans emploi  [_] Autre (_______________________) | | | |
| **B11** | Quelle est votre religion ? | | [_] Christianisme [_] Islam [_] Animisme  [_] Autre (_________) | | | |
| **B12** | Quel est votre revenu mensuel (en FCFA) ? | | [_]   50.000 [_] 50.001 – 100.000 [_] 100.001 – 200.000 [_] > 200.000 | | | |
| **B13** | Quel est le revenu mensuel de votre ménage (en FCFA) ? | | [_]   50.000 [_] 50.001 – 100.000 [_] 100.001 – 200.000 [_] > 200.000 | | | |
| **B14** | Combien d’enfants vivants avez-vous ? | | [____] enfants | | | |
| **B15** | Combien d’enfants y a-t-il dans le ménage ? | | [____] enfants | | | |
|  |  | |  | | | |
| 1. **CONNAISSANCES SUR LE CANCER DU COL DE L’UTERUS** | | | | | | |
| **C1** | Avez-vous déjà entendu parler du cancer du col de l’utérus ? | | [_] Oui [_] Non | | | |
|  | *Si « C1 = Oui », continuer le remplissage à partir de la question C2*  *Si « C1 = Non », passer à la rubrique « D - Dépistage du cancer du col de l’utérus et motivations »* | | | | | |
| **C2** | Quel est l’agent responsable du cancer du col de l’utérus ? | | [_] HPV (Human Papilloma Virus)  [_] VIH (Virus de l’Immunodéficience Humaine)  [_] Autre(s) (________________________________________________) | | | |
| **C3** | Quels sont les facteurs de risque favorisant une infection au HPV ? | | [_] Précocité des rapports sexuels  [_] Rapports sexuels non protégés  [_] Multiplicité des partenaires sexuels  [_] Multiparité  [_] Échange des objets souillés  [_] Autre(s) (________________________________________________) | | | |
| **C4** | Quelles sont les personnes les plus à risque de développer un cancer du col de l’utérus ? | | [_] Les personnes infectées par le VIH  [_] Les personnes immunodéprimées  [_] Les personnes qui fument  [_] Autre(s) (________________________________________________) | | | |
| **C5** | Le cancer du col de l’utérus peut-il se prévenir ? | | [_] Oui [_] Non | | | |
|  | *Si « C5 = Oui », continuer le remplissage à partir la question C6*  *Si « C5 = Non », passer à la question C7* | | | | | |
| **C6** | Comment peut se prévenir le cancer du col de l’utérus (plusieurs réponses possibles) ? | | [_] Dépistage  [_] Vaccination contre le HPV  [_] Protection lors des rapports sexuels  [_] Rapports sexuels avec un nombre limité de partenaires  [_] Autre (_______________________________) | | | |
| **C7** | Le cancer du col de l’utérus peut-il se guérir ? | | [_] Oui [_] Non | | | |
|  |  | |  | | | |
| 1. **DEPISTAGE DU CANCER DU COL DE L’UTERUS ET APTITUDES** | | | | | | |
|  | **En tant que personnel de santé, comment vous sentez-vous vis-à-vis des tâches suivantes, relativement au dépistage du cancer du col de l’utérus à domicile ?** | | | | | |
| **D1** | Réaliser le counseling pré-test | | | | [_] Pas du tout à l’aise [_] Pas à l’aise  [_] Neutre  [_] A l’aise [_] Tout à fait à l’aise | |
| **D2** | Réaliser le counseling post-test | | | | [_] Pas du tout à l’aise [_] Pas à l’aise  [_] Neutre  [_] A l’aise [_] Tout à fait à l’aise | |
| **D3** | Expliquer la procédure du dépistage | | | | [_] Pas du tout à l’aise [_] Pas à l’aise  [_] Neutre  [_] A l’aise [_] Tout à fait à l’aise | |
| **D4** | Répondre aux questions posées sur le cancer du col de l’utérus | | | | [_] Pas du tout à l’aise [_] Pas à l’aise  [_] Neutre  [_] A l’aise [_] Tout à fait à l’aise | |
| **D5** | Répondre aux questions posées sur le dépistage du cancer du col de l’utérus | | | | [_] Pas du tout à l’aise [_] Pas à l’aise  [_] Neutre  [_] A l’aise [_] Tout à fait à l’aise | |
| **D6** | Conseiller adéquatement une femme ayant un test positif | | | | [_] Pas du tout à l’aise [_] Pas à l’aise  [_] Neutre  [_] A l’aise [_] Tout à fait à l’aise | |
| **D7** | Transporter avec vous des échantillons de prélèvement vaginal | | | | [_] Pas du tout à l’aise [_] Pas à l’aise  [_] Neutre  [_] A l’aise [_] Tout à fait à l’aise | |
| **D8** | Prodiguer aux femmes des explications claires sur les modalités/procédures de réalisation du test | | | | [_] Pas du tout à l’aise [_] Pas à l’aise  [_] Neutre  [_] A l’aise [_] Tout à fait à l’aise | |
| **D9** | Prodiguer aux femmes des conseils si votre test est positif | | | | [_] Pas du tout à l’aise [_] Pas à l’aise  [_] Neutre  [_] A l’aise [_] Tout à fait à l’aise | |
| **D10** | Prodiguer aux femmes des explications claires sur la prise en charge d’un cas positif | | | | [_] Pas du tout à l’aise [_] Pas à l’aise  [_] Neutre  [_] A l’aise [_] Tout à fait à l’aise | |
|  |  | |  | | | |
| 1. **FAISABILITE DU DEPISTAGE PAR ET SELON LES PROFESSIONNELS DE SANTE** | | | | | | |
|  | **Que pensez-vous des dispositions suivantes, quant à leur potentiel dans l’amélioration ou la facilitation de la mise en œuvre du dépistage à domicile pour les femmes ?** | | | | | |
| **E1** | Pensez-vous que l’auto-prélèvement à domicile puisse être proposé en communauté par les ARC seuls ? | | | | [_] Pas du tout à l’aise [_] Pas à l’aise  [_] Neutre  [_] A l’aise [_] Tout à fait à l’aise | |
|  | **Afin d’optimiser la mise en œuvre du dépistage à domicile, selon vous :** | | | | | |
| **E2** | Quelle période de l’année est plus indiquée pour proposer le dépistage à domicile ? | | [_] Saison des pluies [_] Saison sèche [_] Autant la saison sèche que la saison des pluies | | | |
| **E3** | Quels jours de la semaine sont plus indiqués pour proposer le dépistage à domicile ? | | [_] Les jours ouvrables [_] Les week-ends [_] Les jours sacrés | | | |
| **E4** | Quel moment de la journée est plus indiqué pour proposer le dépistage à domicile ? | | [_] Le matin [_] Dans l’après-midi [_] La soirée | | | |
| **E5** | Quel(s) moyen(s), pour la communication des résultats aux femmes, est (sont) indiqué(s) ? | | [_] Appel [_] Message [_] Face à face | | | |
| **E6** | Quel(s) lieu(x), pour la communication des résultats, vous semble(nt) indiqué(s) ? | | [_] Hôpital Régional Annexe [_] Maison [_] Centre de santé du village | | | |
| **E7** | Quel(s) moyen(s) vous semble(nt) adapté(s) pour la communication sur le dépistage à domicile ? | | [_] Média-radio [_] Communiqués écrits [_] Mégaphones via ARC [_] Autre(s) | | | |

**Merci pour votre participation**
