# Supplementary material for: Acceptability of home-based HPV self-sampling for cervical cancer screening among users and providers in the West region of Cameroon: a cross-sectional study
Source: BMC Health Serv Res. 2025 Oct 3;25:1303. doi: 10.1186/s12913-025-13467-1 (PMC12495857; doi:10.1186/s12913-025-13467-1)
Supplement: Supplementary file 2 — Supplementary Material 2 [file 12913_2025_13467_MOESM2_ESM.docx]

**Additional file 2: Acceptability of home-based cervical cancer screening per health area**

| Health areas | Attitude | Women (%) | Men (%) | Community leaders (%) | Health care providers (%) | Global (%) | Sample |
| --- | --- | --- | --- | --- | --- | --- | --- |
| Dombouo | Favorable | 32(94,12) | 5(83,33) | 1(50) | 14(93,33) | 52(91,23) | 57 |
|  | Unfavorable | 2(5,88) | 1(16,67) | 1(50) | 1(6,67) | 6(8,77) |  |
| Fiala Foreke | Favorable | 120(85,71) | 21(72,41) | 9(100) | 81(88,04) | 231(85,55) | 270 |
|  | Unfavorable | 20(14,29) | 8(27,59) |  | 11(11,96) | 39(14,45) |  |
| Fokoue | Favorable | 8(88,89) | 2(100) | 2(100) | 5(100) | 17(94,44) | 18 |
|  | Unfavorable | 1(11,11) |  |  |  | 1(5,56) |  |
| Fonakeukeu | Favorable | 21(91,30) | 1(100) | 2(100) | 4(100) | 28(93,33) | 30 |
|  | Unfavorable | 2(8,70) |  |  |  | 2(6,67) |  |
| Fondonera | Favorable | 20(90,91) | 3(100) | 3(75) | 2(100) | 29(93,54) | 31 |
|  | Unfavorable | 2(9,09) |  | 1(25) |  | 2(6,45) |  |
| Fontsa-Toula | Favorable | 7(63,63) |  | 1(100) | 4(100) | 12(75) | 16 |
|  | Unfavorable | 4(36,36) |  |  |  | 4(25) |  |
| Fotetsa | Favorable | 4(100) | 2(100) | 4(100) | 3(100) | 13(100) | 13 |
|  | Unfavorable |  |  |  |  |  |  |
| Ndoh-Djuittitsa | Favorable | 24(85,71) | 2(100) | 2(66,67) | 6(85,71) | 33(82,5) | 40 |
|  | Unfavorable | 4(14,29) |  | 1(33,33) | 1(14,29) | 7(17,5) |  |
| Siteu | Favorable | 23(79,31) | 24(96) | 6(100) | 20(95,24) | 73(90,12) | 81 |
|  | Unfavorable | 6(20,69) | 1(4) |  | 1(4,76) | 8(9,88) |  |
|  | Total favorable | 259 (83,33) | 59 (84,29) | 30 (90,90) | 138 (90,20) |  | 556 |
