# Supplementary material for: Acceptability of home-based HPV self-sampling for cervical cancer screening among users and providers in the West region of Cameroon: a cross-sectional study
Source: BMC Health Serv Res. 2025 Oct 3;25:1303. doi: 10.1186/s12913-025-13467-1 (PMC12495857; doi:10.1186/s12913-025-13467-1)
Supplement: Supplementary file 3 — Supplementary Material 3 [file 12913_2025_13467_MOESM3_ESM.docx]

**Additional file 3: Factors associated to acceptability of home-based CC screening among women, men and HCPs**

**Women**

Factors associated with favorable acceptability of home-based cervical cancer screening among women

|  |  | **Acceptability of home-based CC screening** | | | | | | **Univariate regression** | | | |  |
| --- | --- | --- | --- | --- | --- | --- | --- | --- | --- | --- | --- | --- |
|  | N | Favorable | | | | Unfavorable | | | cOR | 95%CI | p | |
|  |  | n | | (%) | | n | (%) | |  |  |  | |
| **Place of residence** |  | |  | |  |  |  | |  |  |  | |
| Rural | 131 | | 96 | | (73.3) | 35 | (26.7) | | 1 |  |  | |
| Semi urban | 29 | | 20 | | (69.0) | 9 | (31.0) | | 0.8 | (0.3;1.9) | 0.638 | |
| Urban | 140 | | 105 | | (75.0) | 35 | (25.0) | | 1.1 | (0.6;1.9) | 0.747 | |
| **Age** |  | |  | |  |  |  | |  |  |  | |
| [30-40[ years | 183 | | 134 | | (73.2) | 49 | (26.8) | | 1 |  |  | |
| [40-50[ years | 117 | | 87 | | (74.4) | 30 | (25.6) | | 1.06 | (0.6;1.8) | 0.828 | |
| **Marital status** |  | |  | |  |  |  | |  |  |  | |
| In a relationship | 253 | | 188 | | (74.3) | 65 | (25.7) | | 1.2 | (0.6;2.4) | 0.559 | |
| Single | 47 | | 33 | | (70.2) | 14 | (29.8) | | 1 |  |  | |
| **Instruction level** |  | |  | |  |  |  | |  |  |  | |
| Not been to school/primary | 94 | | 63 | | (67.0) | 31 | (33.0) | | 1 |  |  | |
| Secondary | 153 | | 120 | | (78.4) | 33 | (21.6) | | 1.8 | (1.0;3.2) | 0.048 | |
| Higher | 53 | | 38 | | (71.7) | 15 | (28.3) | | 1.2 | (0.6;2.6) | 0.557 | |
| **Religion** |  | |  | |  |  |  | |  |  |  | |
| Animist | 36 | | 26 | | (72.2) | 10 | (27.8) | | 0.9 | (0.4;2.0) | 0.835 | |
| Atheist/others | 23 | | 17 | | (73.9) | 6 | (26.1) | | 1.0 | (0.4;2.6) | 0.995 | |
| Monotheism | 241 | | 178 | | (73.9) | 63 | (26.1) | | 1 |  |  | |
| **Monthly income** |  | |  | |  |  |  | |  |  |  | |
| ≤ 50 000 XAF | 251 | | 182 | | (72.5) | 69 | (27.5) | | 1 |  |  | |
| > 50 000 XAF | 46 | | 38 | | (82.6) | 8 | (17.4) | | 1.8 | (0.8;4.0) | 0.155 | |
| **Household monthly income** |  | |  | |  |  |  | |  |  |  | |
| ≤ 100 000 XAF | 213 | | 157 | | (73.7) | 56 | (26.3) | | 1 |  |  | |
| > 100 000 XAF | 67 | | 51 | | (76.1) | 16 | (23.9) | | 1.1 | (0.6;2.1) | 0.694 | |
| **Level of knowledge about CC** |  | |  | |  |  |  | |  |  |  | |
| Adequate | 188 | | 139 | | (73.9) | 49 | (26.1) | | 1.0 | (0.6;1.8) | 0.891 | |
| Insufficient | 112 | | 82 | | (73.2) | 30 | (26.8) | | 1 |  |  | |
| **Ever had CC screening** |  | |  | |  |  |  | |  |  |  | |
| No | 201 | | 147 | | (73.1) | 54 | (26.9) | | 1 |  |  | |
| Yes | 99 | | 74 | | (74.7) | 25 | (25.3) | | 1.1 | (0.6;1.9) | 0.766 | |

**Men / close relatives**

Factors associated to favorable acceptability of home-based cervical cancer screening among men and close relatives

|  |  | **Acceptability of home-based CC screening** | | | | **Univariate regression** | | |
| --- | --- | --- | --- | --- | --- | --- | --- | --- |
|  | N | Favorable | | Unfavorable | | cOR | 95%CI | P |
|  |  | n | (%) | n | (%) |  |  |  |
| **Place of residence** |  |  |  |  |  |  |  |  |
| Rural | 16 | 10 | (62.5) | 6 | (37.5) | 1 |  |  |
| Semi urban | 25 | 13 | (52.0) | 12 | (48.0) | 0.6 | (0.2;2.3) | 0.510 |
| Urban | 29 | 17 | (58.6) | 12 | (41.4) | 0.8 | (0.2;3.0) | 0.799 |
| **Age** |  |  |  |  |  |  |  |  |
| < 40 years | 16 | 8 | (50.0) | 8 | (50.0) | 0.5 | (0.1;1.8) | 0.261 |
| [40-60[ years | 32 | 17 | (53.1) | 15 | (46.9) | 0.5 | (0.2;1.6) | 0.271 |
| ≥ 60 years | 22 | 15 | (68.2) | 7 | (31.8) | 1 |  |  |
| **Instruction level** |  |  |  |  |  |  |  |  |
| Not been to school/primary | 27 | 16 | (59.3) | 11 | (40.7) | 1 |  |  |
| Secondary | 33 | 18 | (54.5) | 15 | (45.5) | 0.8 | (0.3;2.3) | 0.825 |
| Higher | 9 | 6 | (66.7) | 3 | (33.3) | 1.4 | (0.3;6.7) | 0.694 |
| **Occupation** |  |  |  |  |  |  |  |  |
| Jobless/other | 18 | 10 | (55.6) | 8 | (44.4) | 1 |  |  |
| Formal sector | 18 | 10 | (55.6) | 8 | (44.4) | 1.0 | (0.3;3.7) | 1.000 |
| Informal sector | 34 | 20 | (58.8) | 14 | (41.2) | 1.1 | (0.4;3.6) | 0.821 |
| **Religion** |  |  |  |  |  |  |  |  |
| Animism-other | 13 | 7 | (53.8) | 6 | (46.2) | 1 |  |  |
| Christianism | 57 | 33 | (57.9) | 24 | (42.1) | 1.2 | (0.3;3.9) | 0.790 |
| **Monthly income** |  |  |  |  |  |  |  |  |
| ≤ 50 000 XAF | 46 | 25 | (54.3) | 21 | (45.7) | 1 |  |  |
| > 50 000 XAF | 20 | 12 | (60.0) | 8 | (40.0) | 1.3 | (0.4;3.7) | 0.671 |
| **Knowledge about CC** |  |  |  |  |  |  |  |  |
| Insufficient | 25 | 15 | (60.0) | 10 | (40.0) | 1 |  |  |
| Adequate | 45 | 25 | (55.6) | 20 | (44.4) | 0.8 | (0.3;2.2) | 0.719 |

**HCPs**

Factors associated to favorable acceptability of home-based cervical cancer screening among health care providers

|  |  | **Acceptability of home-based CC screening** | | | | **Univariate regression** | | |  |
| --- | --- | --- | --- | --- | --- | --- | --- | --- | --- |
|  | N | Favorable | | Unfavorable | | cOR | 95%CI | p | |
|  |  | n | (%) | n | (%) |  |  |  | |
| **Place of residence** |  |  |  |  |  |  |  |  | |
| Rural | 40 | 36 | (90.0) | 4 | (10.0) | 1 |  |  | |
| Semi urban | 21 | 19 | (90.5) | 2 | (9.5) | 1.1 | (0.2;6.3) | 0.953 | |
| Urban | 92 | 79 | (85.9) | 13 | (14.1) | 0.7 | (0.2;2.2) | 0.517 | |
| **Age** |  |  |  |  |  |  |  |  | |
| [20-30[ years | 55 | 45 | (81.8) | 10 | (18.2) | 0.7 | (0.2;2.3) | 0.526 | |
| [30-40[ years | 67 | 62 | (92.5) | 5 | (7.5) | 1.8 | (0.5;7.4) | 0.391 | |
| ≥40 years | 31 | 27 | (87.1) | 4 | (12.9) | 1 |  |  | |
| **Marital status** |  |  |  |  |  |  |  |  | |
| In a relationship | 93 | 83 | (89.2) | 10 | (10.8) | 1.5 | (0.6;3.8) | 0.439 | |
| Single | 60 | 51 | (85.0) | 9 | (15.0) | 1 |  |  | |
| **Instruction level** |  |  |  |  |  |  |  |  | |
| Not been to school/secondary | 45 | 39 | (86.7) | 6 | (13.3) | 1 |  |  | |
| Higher | 108 | 95 | (88.0) | 13 | (12.0) | 1.1 | (0.4;3.2) | 0.825 | |
| **Function** |  |  |  |  |  |  |  |  | |
| Nurse | 104 | 93 | (89.4) | 11 | (10.6) | 1.6 | (0.5;5.6) | 0.451 | |
| Medical doctor | 11 | 9 | (81.8) | 2 | (18.2) | 0.9 | (0.1;5.5) | 0.872 | |
| Midwife | 13 | 11 | (84.6) | 2 | (15.4) | 1.0 | (0.2;6.6) | 0.961 | |
| Other | 25 | 21 | (84.0) | 4 | (16.0) | 1 |  |  | |
| **Work experience** |  |  |  |  |  |  |  |  | |
| ≤ 5 years | 79 | 70 | (88.6) | 9 | (11.4) | 1 |  |  | |
| > 5 years | 70 | 61 | (87.1) | 9 | (12.9) | 0.9 | (0.3;2.3) | 0.784 | |
| **Level of knowledge about CC** |  |  |  |  |  |  |  |  | |
| Insufficient | 9 | 6 | (66.7) | 3 | (33.3) | 1 |  |  | |
| Adequate | 144 | 128 | (88.9) | 16 | (11.1) | 4.0 | (0.9;17.6) | 0.066 | |
