# Supplementary material for: Acceptability of home-based HPV self-sampling for cervical cancer screening among users and providers in the West region of Cameroon: a cross-sectional study
Source: BMC Health Serv Res. 2025 Oct 3;25:1303. doi: 10.1186/s12913-025-13467-1 (PMC12495857; doi:10.1186/s12913-025-13467-1)
Supplement: Supplementary file 4 — Supplementary Material 4 [file 12913_2025_13467_MOESM4_ESM.docx]

**Additional file 4:** **Items of acceptability for women (N=300), men and close relatives (N=70), community leaders (N=33) and HCPs (N=153)**

**Women, men and community leaders**

| **Group** | **Women (N=300)** | | **Men (N=70)** | | **Community Leaders (N=33)** | | | |
| --- | --- | --- | --- | --- | --- | --- | --- | --- |
| **Items** | **n** | **%** | **n** | **%** | | **n** | **%** |  |
| I would not be embarrassed if I/if my wife/if a woman from my community did the self-sampling at home | 210 | (70.0) | 58 | (82.9) | | 30 | (90.9) |  |
| I am not afraid that my wife would not perform the self-sampling correctly at home | 205 | (68.3) | 51 | (72.9) | | 22 | (66.7) |  |
| I am not afraid of what others at home might think about my wife’s status/result | 235 | (78.3) | 61 | (87.1) | | 30 | (90.9) |  |
| I am not afraid that she would make others at home uncomfortable | 222 | (74.0) | 59 | (84.3) | | 27 | (81.8) |  |
| I am comfortable with the fact that my wife receives education on cervical cancer at home | 273 | (91.0) | 61 | (87.1) | | 25 | (75.8) |  |
| The distance between our home and the nearest hospital offering cervical cancer screening is not long and does not discourage my wife from going to get screened | 213 | (71.0) | 46 | (65.7) | | 24 | (72.7) |  |
| The quickness of the screening process encourages me to encourage my wife to do it | 204 | (68.0) | 47 | (67.1) | | 15 | (45.5) |  |
| The house is clean and hygienic enough to perform the screening | 168 | (56.0) | 43 | (61.4) | | 17 | (51.5) |  |
| Other members of the household cannot discourage or prevent my wife from doing the screening at home | 265 | (88.3) | 8 | (11.4) | | 14 | (42.4) |  |
| Even if he knows, my partner cannot discourage or prevent me from doing the screening at home | 259 | (86.3) | - | - | | - | - |  |
| ***^1^****: asked to women;* ***^2^****: asked to men/close relatives;* ***^3^****: asked to community leaders; LC: community leaders*  *This adaptation is to be considered for all the questions in this section.*  *For presentation and interpretation purposes, questions are reformulated from the original (from the original formulation in the questionnaires), to fit the modalities “agree” and “totally agree”.* | | | | | | | | |

**HCPs (N=153)**

| **Items** | **n** | **(%)** |
| --- | --- | --- |
| Conduct pre-test counseling | 123 | (80.4) |
| Conduct post-test counseling | 121 | (79.1) |
| Explain the screening procedure | 127 | (83) |
| Answer questions about cervical cancer | 137 | (89.5) |
| Answer questions about cervical cancer screening | 134 | (87.6) |
| Properly counsel a woman with a positive HPV test result | 121 | (79.1) |
| Transport vaginal sample specimens with you | 102 | (66.7) |
| Provide women with clear explanations on how to perform the test | 138 | (90.2) |
| Provide women with advice if the test result is positive | 146 | (95.4) |
| Provide women with clear explanations on how to manage a positive case | 146 | (95.4) |
